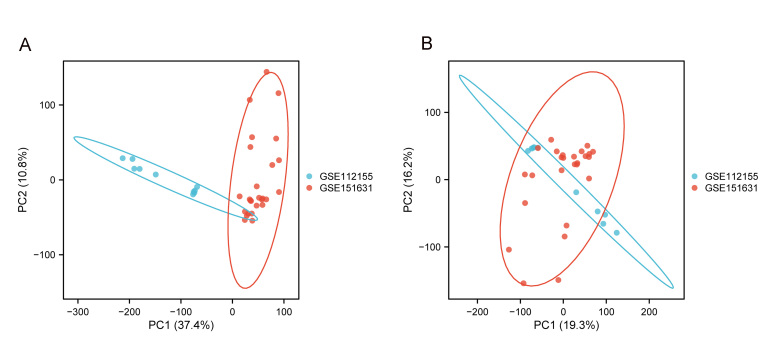# Identification and validation of autophagy-related genes in keratoconus and their correlation with immune infiltration

**DOI:** 10.1097/MD.0000000000048985

**Published:** 2026-05-29

**Authors:** Sutong Li, Gang Wang, Jing Chen, Naiyang Li

**Affiliations:** a Guangdong Medical University, Zhanjiang, Guangdong, China; b Zhongshan City People’s Hospital, Zhongshan, Guangdong, China.

**Keywords:** autophagy-related genes, immune cell infiltration, keratoconus, MAPK signaling pathway, oxidative stress

## Abstract

Keratoconus (KCN) is a bilateral and asymmetric cornea disease. Autophagy plays an important role in homeostasis by protecting cells against stress. However, the roles of autophagy-related genes (ARGs) in KCN remains unclear. Hence, this study aimed to identify the signatures of ARGs of KCN and explore their correlation with immune infiltration. Transcriptional data and clinical information of patients with KCN were downloaded from the profile data GSE112155 and GSE151631. Functional analysis was used to reflect the biological functions, and weighted gene co-expression network analysis was applied to excavate co-expression modules of autophagy-related expression patterns. Moreover, gene set enrichment and variation analyses were performed for pathway analysis. Consensus clustering analysis was used to cluster different molecular subtypes on the basis of gene expression profiles of KCN-specific ARGs. Single-sample gene set enrichment analysis was employed to calculate separate enrichment scores for each immunocyte between KCN and healthy samples. Finally, hub genes were verified by real-time quantitative polymerase chain reaction. We first identified 14 ARGs differentially expressed between patients with KCN and controls using NetworkAnalyst. Nine overlapped genes (*BNIP3*, *CDKN1A*, *DDIT3*, *FOS*, *HSPA5*, *MAPK8IP1*, *MYC*, *PPP1R15A*, and *VEGFA*) (*P* < .05) were identified using a random forest model. The MAPK signaling pathway, apoptosis, FoxO signaling pathway, and protein processing in endoplasmic reticulum signaling were mainly involved. The weighted gene co-expression network analysis classified the genes into 12 distinct modules. The MEturquoise (correlation = 0.51), MEpink (correlation = 0.535) were significantly positively correlated with KCN, whereas the MEyellow (correlation = ‐0.776) and MEgreen (correlation = ‐0.664) were negatively correlated. Single-sample gene set enrichment analysis showed a close interaction between immune cell infiltration and the development of KCN. Finally, all the 9 hub genes except VEGFA were significantly downregulated (*P *< .05) using real-time quantitative polymerase chain reaction. We described the signatures of ARGs in KCN, the distribution of immune cells between KCN patients and the control, and the correlation between hub genes related to autophagy and KCN disease. This demonstrates the potential roles of autophagy mechanisms and the immune response in KCN, providing a novel insight into understanding the pathogenesis of KCN and potential treatment targets.

## 1. Introduction

Keratoconus (KCN) is a chronic, progressive, and degenerative disorder characterized by thinning and asymmetrical conical protrusion of the cornea that can lead to severe visual impairment due to refractive errors and irregular astigmatism and usually occurs in adolescents and young adults.^[1,2]^ Its prevalence varies widely in different populations, ranging from 0.02/100,000 to 479/100,000 population.^[3]^ Recent studies have reported a increased trend from 0.03% to 0.04% in United States^[4]^ and one of the highest prevalence with 3.4% in the world from the Raine Study.^[5]^ In our region, previous studies have reported an incidence among Asians up to 32.3/100,000,^[6]^ and an incidence of 0.9 ± 0.2% in 3468 Chinese individuals aged 50 years or older.^[7]^ Therefore, exploration of the pathogenesis of KCN is necessary for early detection, and diagnosis and effective management of KCN.

Numerous previous studies have revealed that genetic and environmental factors are involved in KCN susceptibility.^[8]^ Environmental factors, for example, oxidative stress from ultraviolet light-induced damage, allergy, asthma, and eye rubbing have been suggested as aggravating factors in patients with KCN.^[9]^ Results of families segregation analysis, linkage analysis, next generation sequencing studies, and genome-wide association studies (GWAS) have also indicated that genetic factors are involved in the pathogenesis of KCN, and several candidate genes have been identified, including the key candidate genes visual system homeobox 1 (*VSX1*)^[10]^ and superoxide dismutase 1 (*SOD1*)^[11]^ genes, although this remains controversial. Further, the specific pathogenesis of KCN remains poorly understood.

KCN is primary caused by stromal layer abnormality with epithelial lesions often secondary to mechanical changes in stromal layer. Collagen degradation due to the release of proteolytic enzymes resulted in the thinning of the cornea.^[12]^ Oxidative stress is a key factor contributing to collagen degradation during KCN.^[13,14]^ Autophagy often occurs in response to oxidative stress, and autophagy induced by oxidative stress, may be a key factor in the pathogenesis of KCN. Previous studies demonstrated oxidative stress induced dysregulation of autophagy in the corneal epithelium of patients with KCN.^[15,16]^ However, the mechanisms underlying autophagy in KCN remain unclear. Recent studies have shown alterations in the expression of molecules involved in the inflammatory and immune processes of KCN.^[17–19]^ Autophagy also influences immune response and inflammation.^[20–22]^ Although emerging evidence demonstrating immune cell infiltration in KCN has been reported,^[18,19]^ the correlation between immune cell infiltration and autophagy affecting the KCN process remains largely unknown. Therefore, we aimed to explore the association between autophagy-related genes (ARGs) and KCN, the potential role of ARGs in KCN, and the correlation between ARGs and immune cells. Our findings may provide new insights into the molecular mechanisms of KCN and highlight potential therapeutic targets.

## 2. Materials and methods

### 2.1. Data acquisition and processing

**Figure 1.**
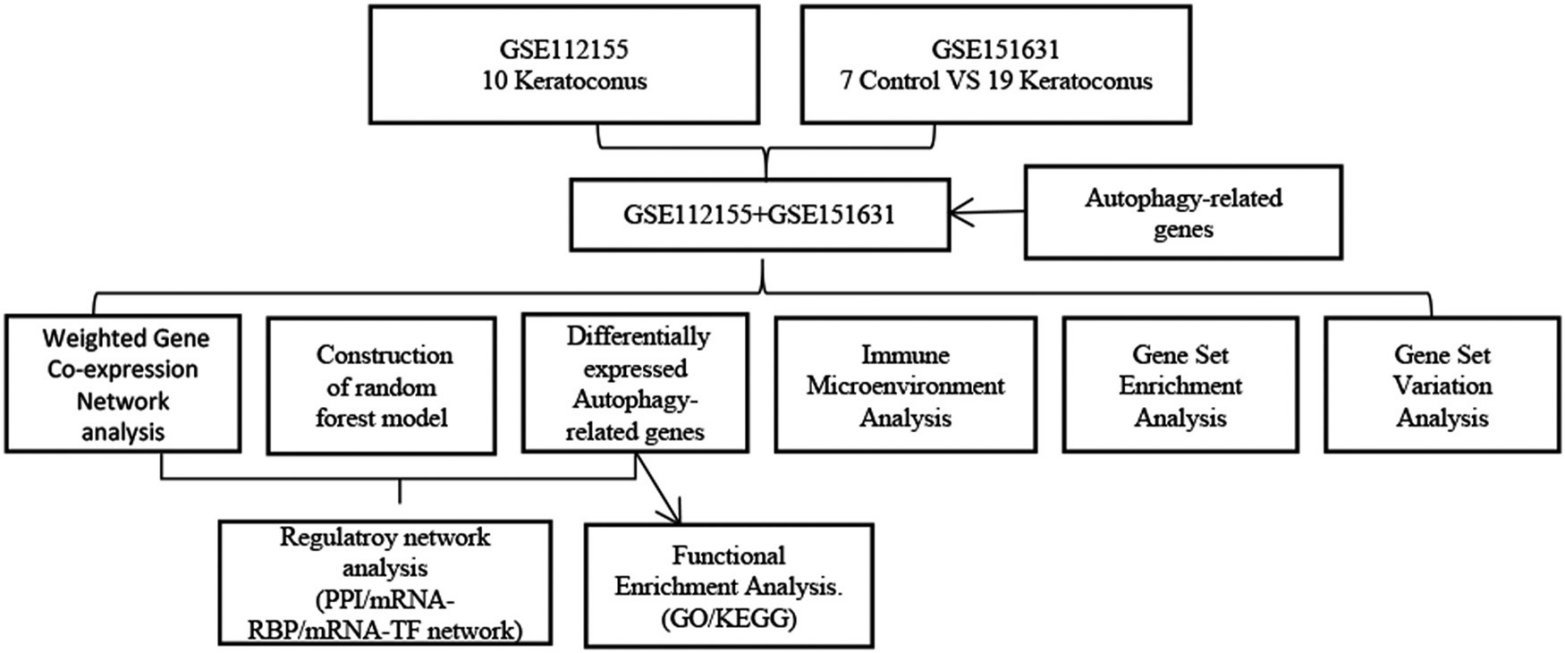
Study flowchart.

A flowchart of this study, which aimed to identify the signatures of ARGs in KCN and explore their correlation with immune infiltration, is shown in Figure 1. We retrieved and downloaded 2 datasets, GSE112155^[23]^ and GSE151631,^[24]^ from the Gene Expression Omnibus database. GSE112155 included 10 samples from KCN cases and 10 samples from myopic controls. The 10 samples from myopic controls were excluded in this study. Control corneal epithelial tissues were collected from patients undergoing laser photorefractive keratectomy for mild myopia <6 diopters. Prior to the laser ablation, the cornea is prepared by the manual removal of the central 8 mm of epithelium. KCN epithelial tissue was collected from patients undergoing collagen cross-linking to halt the progression of the disease. GSE151631 containsthe expression profiles of 26 samples, including 19 patients with KCN cases and 7 healthy controls. KCN patients are African American ancestry (KC) from Baltimore (17–69 years old) and of Middle Eastern ancestry from Saudi Arabia (18–36 years old). Controls samples are from deceased African American donors (25–75 years old). Experiments were conducted on a GPL18573 Illumina NextSeq 500 (*Homo sapiens*) and a GPL16791 Illumina HiSeq 2500 (*H sapiens*). Corneal samples were collected from patinets with KCN (n = 29) and controls (n = 7). Batch effect removal was performed using ComBat^[25]^ in the sva R package (R Core Team) and the data were pooled for subsequent analysis.

Both GSE112155 and GSE151631 are transcriptome datasets of human corneal tissue from keratoconus patients and healthy controls. These 2 datasets maintain consistency in sampling site and tissue type, with well-defined grouping criteria, demonstrating good biological comparability. Although the sequencing platforms differ, they are both high-throughput RNA sequencing platforms. The sample collection and processing procedures are transparent, and the data quality and formats are highly compatible, thus ensuring the comparability and scientific validity of the combined data analysis. We have employed the ComBat algorithm from the sva package to correct for batch effects in the merged expression matrix. After data integration, we conducted an internal evaluation of its distribution characteristics and differential analysis results, confirming that the impact of batch effects has been significantly reduced while biological differences have been preserved. PCA plots before and after correction are shown in Figure S1, Supplemental Digital Content.

The Human Autophagy Database is a human autophagy-dedicated database containing comprehensive information to date on human genes related to autophagy, from which we extracted 232 ARGs (Table S1, Supplemental Digital Content). The analysis of publicly available datasets (GSE112155 and GSE151631) did not require additional ethics approval as per local guidelines and the original studies obtained appropriate ethical clearance. For the experimental validation part, the studies involving human participants were reviewed and approved by The Clinical Research and Animal Experiment Ethics Committee of Zhongshan People’s Hospital (2025-061).

### 2.2. Identification of differentially expressed ARG genes

NetworkAnalyst (http://www.networkanalyst.ca) is a platform for the visualization of comprehensive gene expression profiling and meta-analysis, including alignment and quantification, differentially expressed gene analysis, protein–protein interaction (PPI), and integrative analysis of multiple datasets.^[26]^ We employed NetworkAnalyst to normalize the data and identify differentially expressed genes (DEGs) in each dataset with thresholds of adjusted *P* < .05 and |log2 fold change| (|log2 FC|) >1. Genes overlapping between ARGs and DEGs were identified. Volcano plots and heat maps were generated using the ggplot2^[27]^ and pheatmap^[28]^ packages of R.

### 2.3. Construction of random forest models

The random forest model, which is an ensemble learning method, is an extension of the decision tree algorithm.^[29]^ The DEGs were screened out as models of the development of KCN disease using the random forest algorithm. “Reverse cumulative distribution of residual” and “boxplot of the residual distribution” were drawn to evaluate the model.

### 2.4. Construction of the interaction network

The GeneMANIA^[30]^ is a repository of gene functions that is available with genomics and proteomics data for the generation of hypotheses, analysis of gene lists, and prioritizing genes for functional assays. The GeneMANIA database was used to predict the interacting proteins of the differentially expressed ARGs and construct PPI networks. TRRUST^[31]^ is a versatile database of transcriptional regulatory networks. It was used to predict the interactions between differentially expressed ARGs and transcription factors (TFs). Here, we predicted the TF related to differentially expressed ARGs using TRRUST and built a messenger-RNA–TF interaction network. The starBase database^[32]^ includes data of micro (mi)-RNA–noncoding-RNA, miRNA–mRNA, RNA binding protein (RBP)–RNA, and RNA–RNA, and we predicted RBP–RNA of differentially expressed ARGs on the basis of the following: Clade (mammal), Genome (human), Assembly (hg19), CLIP-Data (≥5), and pan-Cancer (≥0).

### 2.5. Functional enrichment analysis

ClusterProfiler^[33]^ in R was used for Gene Ontology (GO)^[34]^ functional enrichment analysis. Kyoto Encyclopedia of Genes and Genomes (KEGG)^[35]^ enrichment analysis were performed. Statistical significance was set at *P* < .05 statistically significant. Gene set enrichment analysis (GSEA)^[36]^ was used to determine the enrichment of specific gene sets between patients with KCN and the controls. We also used gene set variation analysis (GSVA)^[37]^ to normalize the expression matrix and determined the pathway activation by estimating the pathway activation scores between KCN and the control group using the R package “limma.”^[38]^ The “C2.all.v7.2.symbols.GMT [Curated]” gene set was downloaded from the MSigDB database^[39]^ for GSEA and GSVA analyses, and the limma package^[40]^ was applied to filter the enrichment pathway with a threshold of |logFC| > 1 and an adjusted *P* < .05.

### 2.6. Weighted gene co-expression network analysis (WGCNA)

Co-expression analysis was performed using the WGCNA R software package.^[41]^ A weighted adjacency matrix was constructed by determining the Pearson correlation coefficients of individual gene pairs; then, appropriate soft thresholding was selected to generate a standardized network, and the matrix was converted into a topological overlap matrix. Additionally, hierarchical cluster analysis was performed, and the dynamic tree-cutting method was used to construct gene dendrograms and identify clusters (modules) of highly correlated genes.

### 2.7. Clustering analysis

Consensus clustering is a hierarchical clustering approach to classifying discovery and assessing the discovered clusters’ robustness and rationality. The Consensus ClusterPlus^[42]^ package in R was used to identify different disease subtypes on the basis of the expression profile data of KCN using consensus clustering methods.

### 2.8. Evaluation of immune cell infiltration

We measured the abundance of immunocytes in KCN and control samples on the basis of corneal tissue expression profiles using the single-sample gene set enrichment analysis.^[36]^ A heat map was constructed to show each sample’s pattern of infiltrating immunocytes in each sample. Correlation analysis between the differentially expressed ARGs and 28 types of infiltrating immune cells was performed.

### 2.9. Real-time quantitative polymerase chain reaction (RT-qPCR)

The studies involving human participants were reviewed. Six keratoconus samples were obtained from pathological keratoconus tissue excised during corneal transplantation, and 6 normal corneas were obtained from the remaining corneal tissue after corneal transplantation. The Clinical Research and Animal Experiment Ethics Committee of Zhongshan People’s Hospital approved the sample collection. (2025-061). Total RNA was extracted from frozen tissues using TRIzol Reagent (Invitrogen). Complementary DNA was synthesized using the PrimeScript RT Master Mix (Takara, Dalian, China). RT-qPCR was performed using the SYBR Premix Ex Taq II kit (Takara) with the CFX96 Real-Time PCR Detection system (Bio-Rad). Relative expression was calculated with normalization to GAPDH values by using the 2^−ΔΔCt^ method. The primers sequences used for RT-qPCR detection are listed in Table S2, Supplemental Digital Content.

### 2.10. Statistical analyses

The *t* test or Mann–Whitney *U* test was used to analyze continuous variables. The Chi-squared test or Fisher exact test was used to analyze categorical variables. The statistical analyses were performed using R software (version 4.1.0) and statistical significance was set at *P* < .05.

## 3. Results

### 3.1. Identification of differentially expressed ARGs

**Figure 2.**
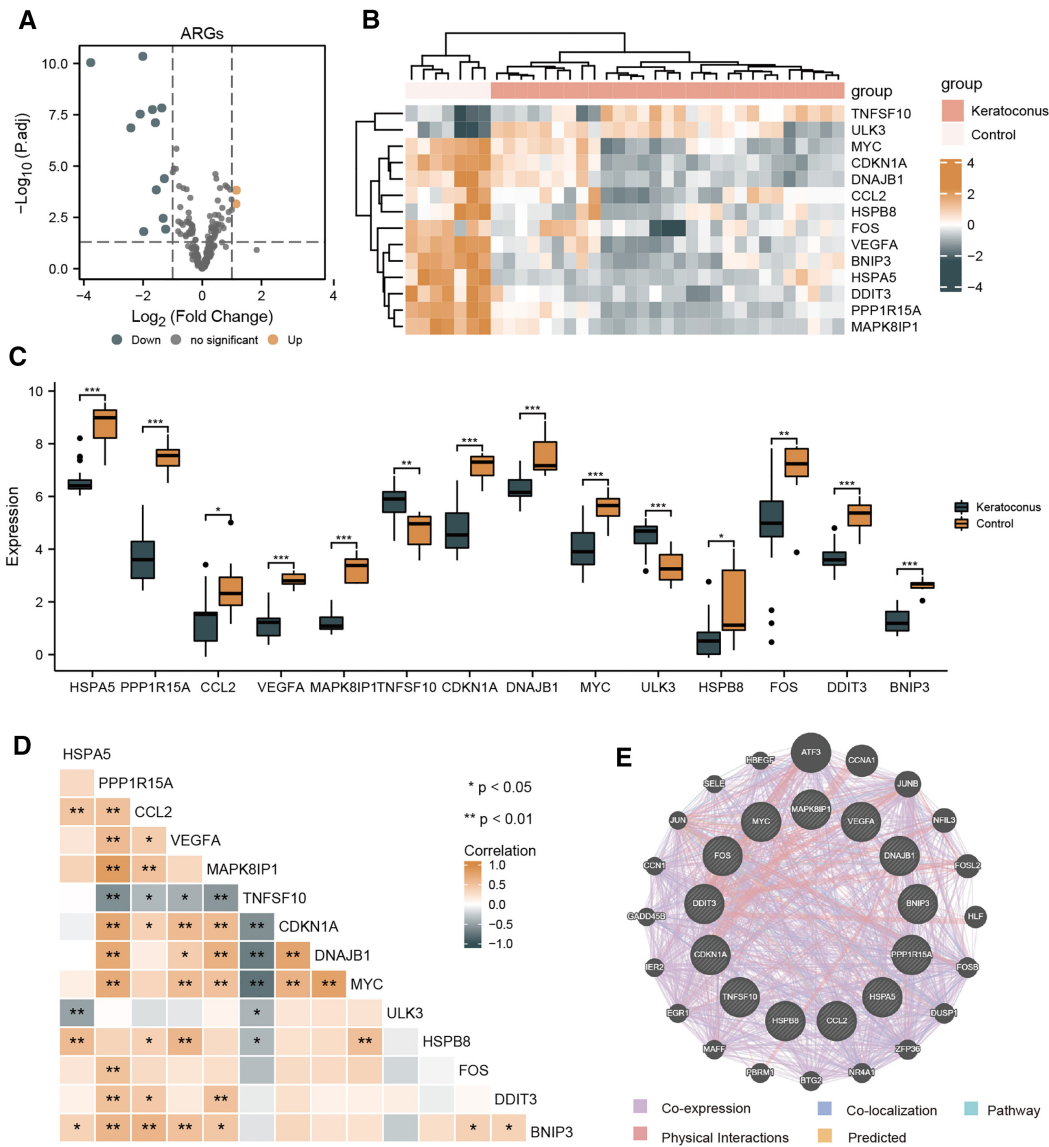
Expression profile of differentially expressed ARGs. Volcano plots (A) and heatmap (B) of differentially expressed ARGs in the KCN and control groups; (C) box diagram of expression of differentially expressed ARGs in KCN and control groups; (D) correlation heatmap of 14 differentially expressed ARGs. Orange represents a positive correlation, and blue represents a negative correlation. The darker the color is, the stronger the correlation is. (E) PPI of differentially expressed ARGs. ARGs = autophagy related genes; KCN = keratoconus; PPI = protein–protein interaction (*P < .05; **P < .01; ***P < .001).

**Figure 3.**
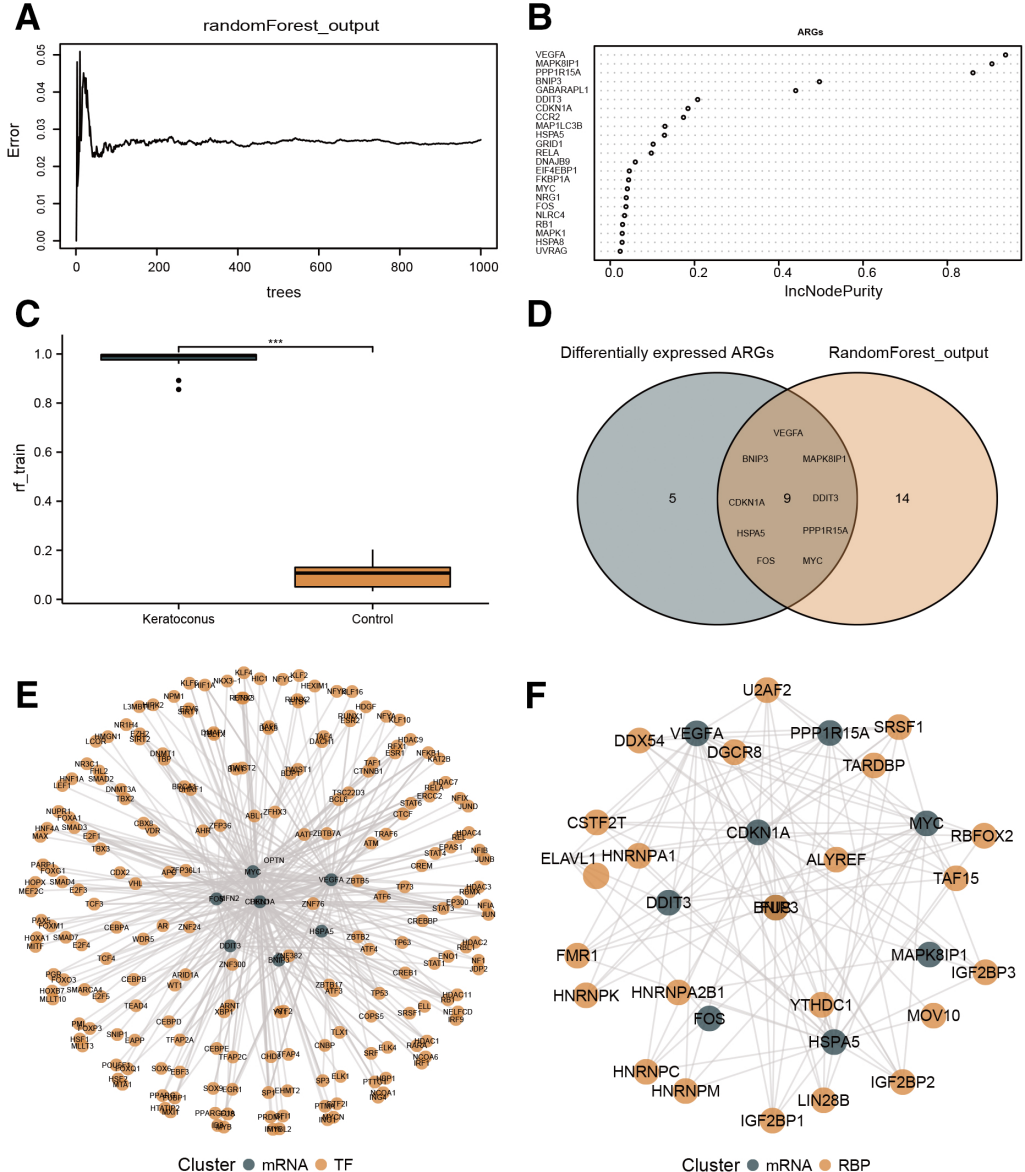
Construction of the random forest model and regulatory network. (A) The relationship between the number of decision trees and the model error. The horizontal axis refers to the number of decision trees, whereas the vertical axis refers to the error rate of the constructed model. (B) The importance index of differentially expressed ARGs in the random forest model. (C) Box diagram of risk scores for the KCN and control groups. (D) Venn diagram of intersected genes from differentially expressed ARGs and random models; (E) mRNA-TF network of differentially expressed ARGs, blue represents mRNA, whereas yellow represents TF. (F) mRNA-RBP network of differentially expressed ARGs; blue represents mRNA, whereas yellow represents RBP. ARGs = autophagy-related genes; KCN = keratoconus; m = messenger; RBP = RNA binding protein; TF = transcriptional factor (***P < .001).

Gene expression profiles and clinical information were obtained from the GSE112155 and GSE151631 databases. A total of 14 differentially expressed ARGs were identified: 2 genes (*ULK3* and *TNFSF10*) that were upregulated and 12 genes (*CCL2*, *DNAJB1*, *HSPB8*, *BNIP3*, *MYC*, *DDIT3*, *VEGFA*, *FOS*, *MAPK8IP1*, *HSPA5*, *CDKN1A*, and *PPP1R15A*) that were downregulated. Volcano plots and heat maps are shown in Figure 2A and B. Box plots were constructed to show the expression of those 14 differentially expressed ARGs in the KCN and control groups (Fig. 2C). The correlation of differentially expressed ARGs in the KCN group is shown in Figure 2D. *HSPA5*, *PPP1R15A*, *CCL2*, *VEGFA*, *MAPK8IP1*, and *TNFSF10* were closely related to each other. The construction of PPI using differentially expressed ARGs is shown in Figure 2E. Additionally, we constructed a random forest model, as shown in Figure 3A and B, and key genes were ranked according to the importance index. The risk score box of KCN and the control group obtained in the random forest model is shown in Figure 3C, and the risk score of KCN was significantly higher than that of the control group. We intersected differentially expressed ARGs with genes from the random model algorithm (Fig. 3D) and ultimately identified 9 genes (*BNIP3*, *CDKN1A*, *DDIT3*, *FOS*, *HSPA5*, *MAPK8IP1*, *MYC*, *PPP1R15A*, and *VEGFA*) that were consistent with the results of NetworkAnalyst. Subsequently, mRNA-TF and mRNA-RBP regulatory networks were constructed for the intersecting genes (Fig. 3E and F).

### 3.2. Functional enrichment of the differentially expressed ARGs

**Figure 4.**
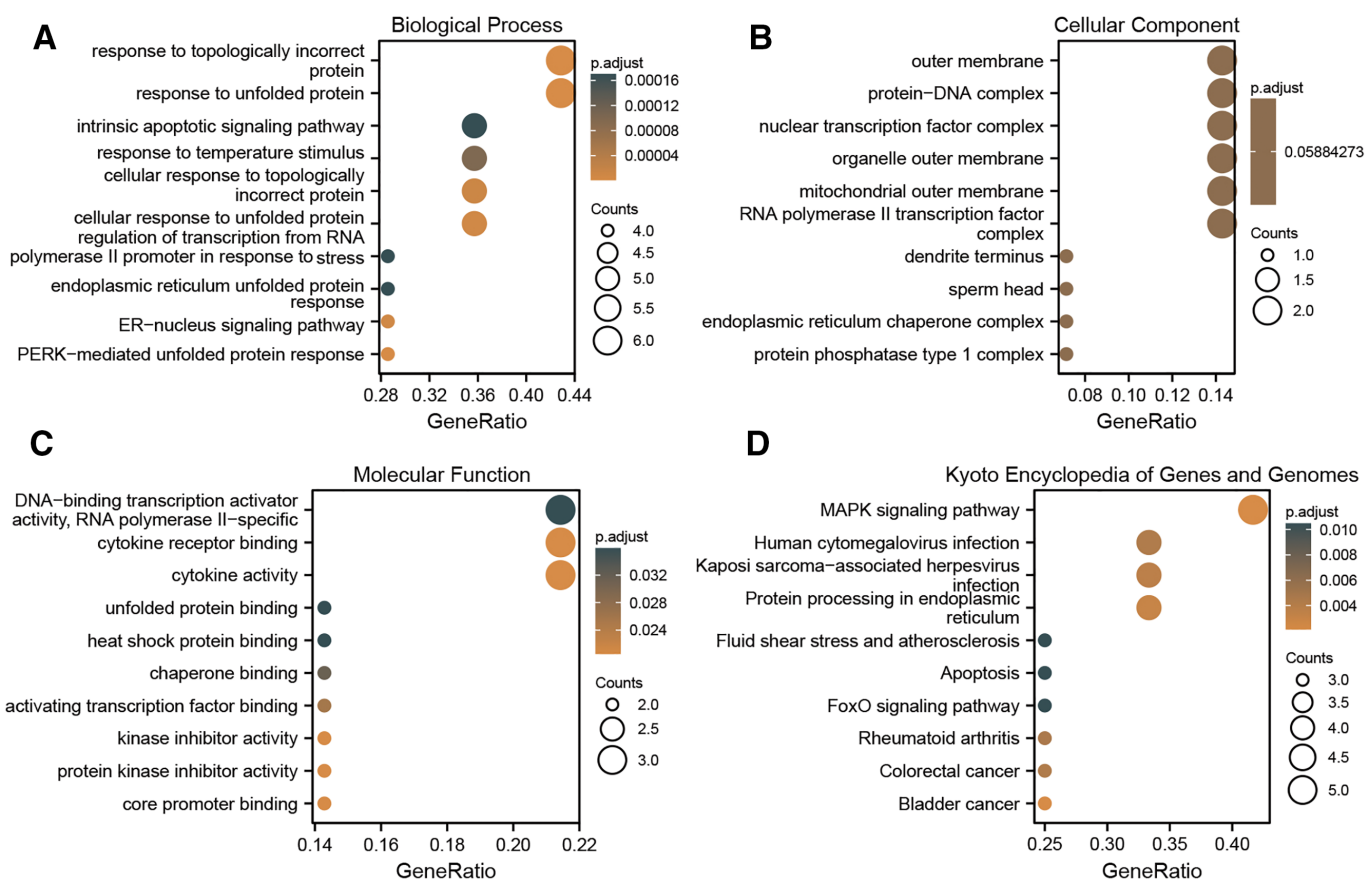
GO and KEGG enrichment analysis of differentially expressed ARGs. (A) Biological process. (B) Cellular component. (C) Molecular function. (D) KEGG. ARGs = autophagy-related genes; GO: Gene Ontology; KEGG = Kyoto Encyclopedia of Genes and Genomes.

To reveal the functional pathways of KCN, we performed GO functional, and KEGG pathway enrichment analyses of the differentially expressed ARGs (Tables S3 and S4, Supplemental Digital Content; Fig. 4). GO analysis of showed that differentially expressed ARGs were mainly involved in response to unfolded protein, response to topologically incorrect protein, intrinsic apoptotic signaling pathway, cellular response to unfolded protein, regulation of transcription from RNA polymerase II, promoter in response to stress, and endoplasmic reticulum (ER)-nucleus signaling pathway (BPs); outer membrane, protein–DNA complex, nuclear TF complex, and organelle outer membrane (CCs); cytokine activity, cytokine receptor binding, unfolded protein binding, heat shock protein binding, chaperone binding (MFs) (Fig. 4A–C; Table S3, Supplemental Digital Content). Moreover, the KEGG results showed that the MAPK signaling pathway, protein processing in ER apoptosis and FoxO signaling pathway were mainly involved (Fig. 4D; Table S4, Supplemental Digital Content).

**Figure 5.**
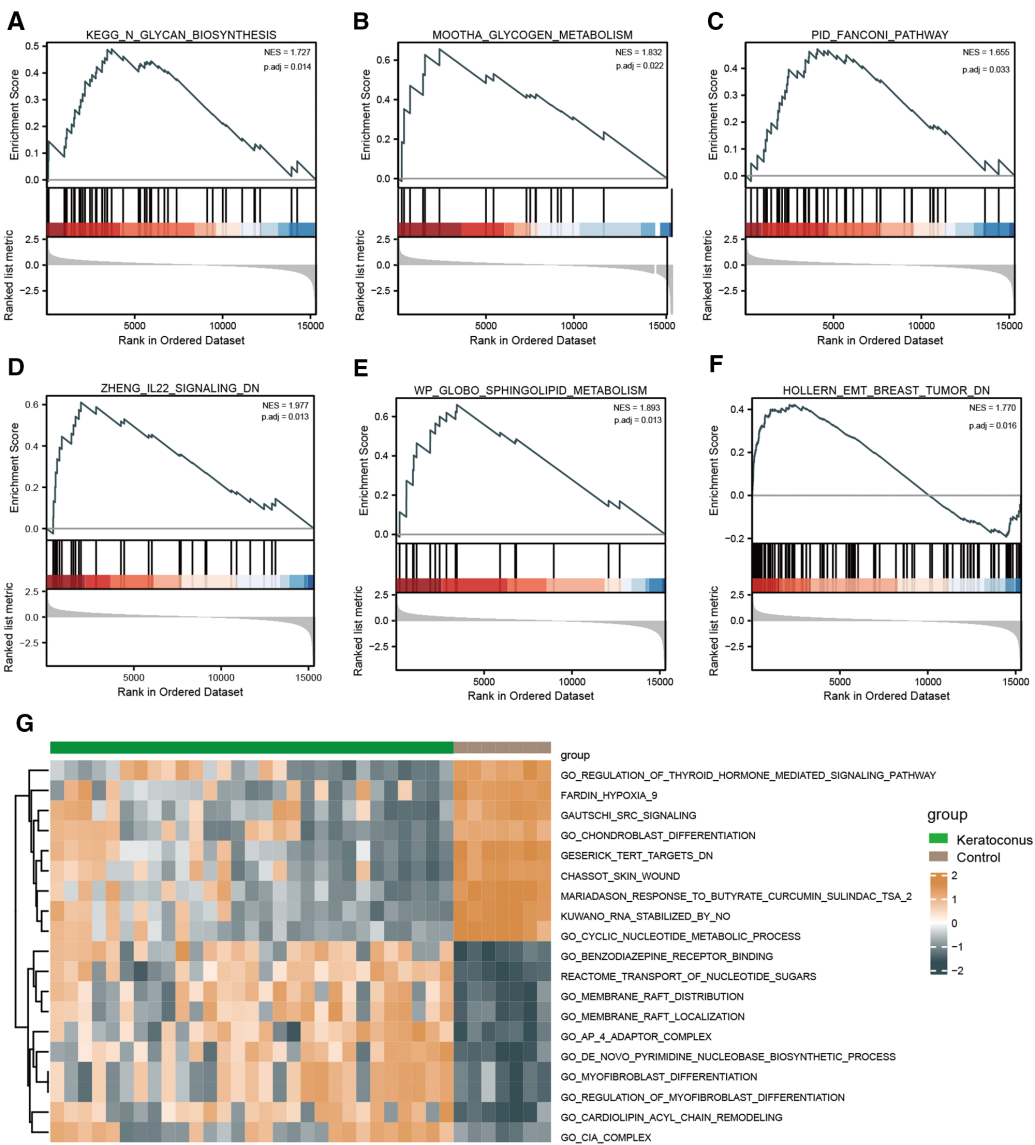
GSEA and GSVA enrichment analyses. (A–F) Enriched pathway of GSEA. (G) GSVA enrichment analysis. GSEA = gene set enrichment analysis; GSVA = gene set variation analysis.

GSEA and GSVA enrichment analyses were also performed. GSEA showed that interleukin-22 signaling, glycan biosynthesis, glycogen metabolism, the Fanconi pathway, sphingolipid metabolism, and EMT breast tumor were significantly enriched in KCN samples (Fig. 5A–F; Table S5, Supplemental Digital Content). The GSVA enrichment analysis results (Fig. 5G; Table S6, Supplemental Digital Content) showed that the process of response to butyrate curcumin sulindac, RNA stabilized by NO, and cyclic nucleotide metabolism were significantly enriched (|logFC| >1, adjusted *P* < .05).

### 3.3. Weighted gene co-expression network analysis

**Figure 6.**
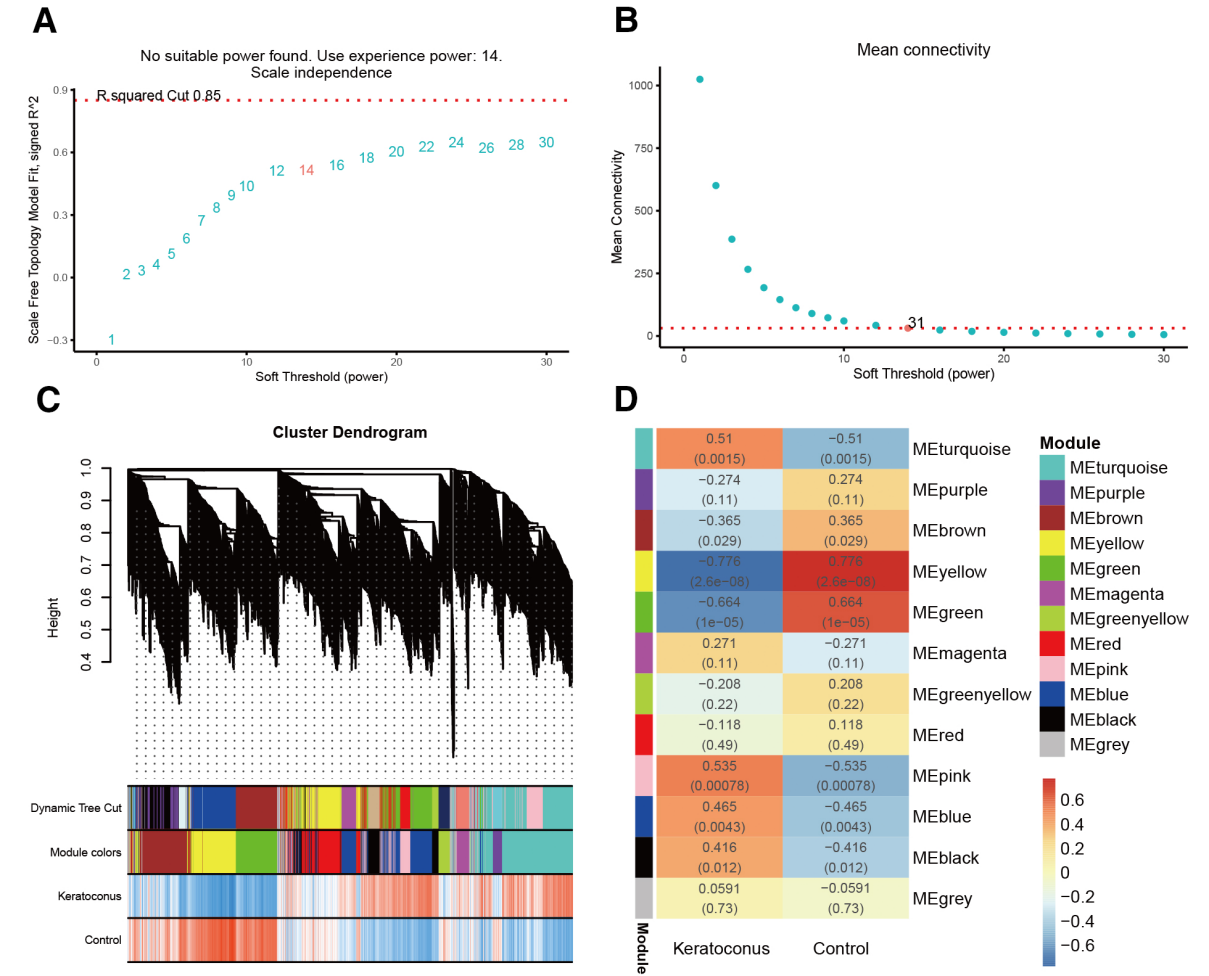
Co-expression modules analysis. (A) Analysis of the scale-free fit index for various soft-thresholding powers (β). (B) Relationship between the mean connectivity and various soft-thresholding powers. (C) Cluster dendrogram of genes from patients with KCN and the control individuals. Each branch in the figure represents 1 gene, and various colors represent different modules. (D) Module–trait relationships in KCN disease. Each cell contains the corresponding correlation and *P*-value. KCN = keratoconus.

The co-expression modules obtained by WGCNA included specific genes with high topological overlap. We used WGCNA, an unsupervised method, to analyze the data of the sample data. A thresholding power of β = 14 (*R*^2^ = 0.85) was used to construct a scale-free network (Fig. 6A and B). A gene dendrogram was obtained using topological overlap-based dissimilarity and the corresponding module colors were assigned. Each colored row underneath the dendrogram denotes a color-coded module containing a set of highly correlated genes. Ultimately, 12 modules were classified using dynamic tree cutting (Fig. 6C). The correlation between each module and samples is shown in Figure 6D. The modules of MEturquoise (correlation = 0.51), MEpink (correlation = 0.535), MEblue (correlation = 0.465), and MEblac (correlation = 0.416) exhibited significant positive correlations with KCN (*R* > 0, *P* < .05), while the MEyellow (correlation = ‐0.776) and MEgreen (correlation = ‐0.664) showed significant negative correlations with KCN (*R* < 0, *P* < .05).

**Figure 7.**
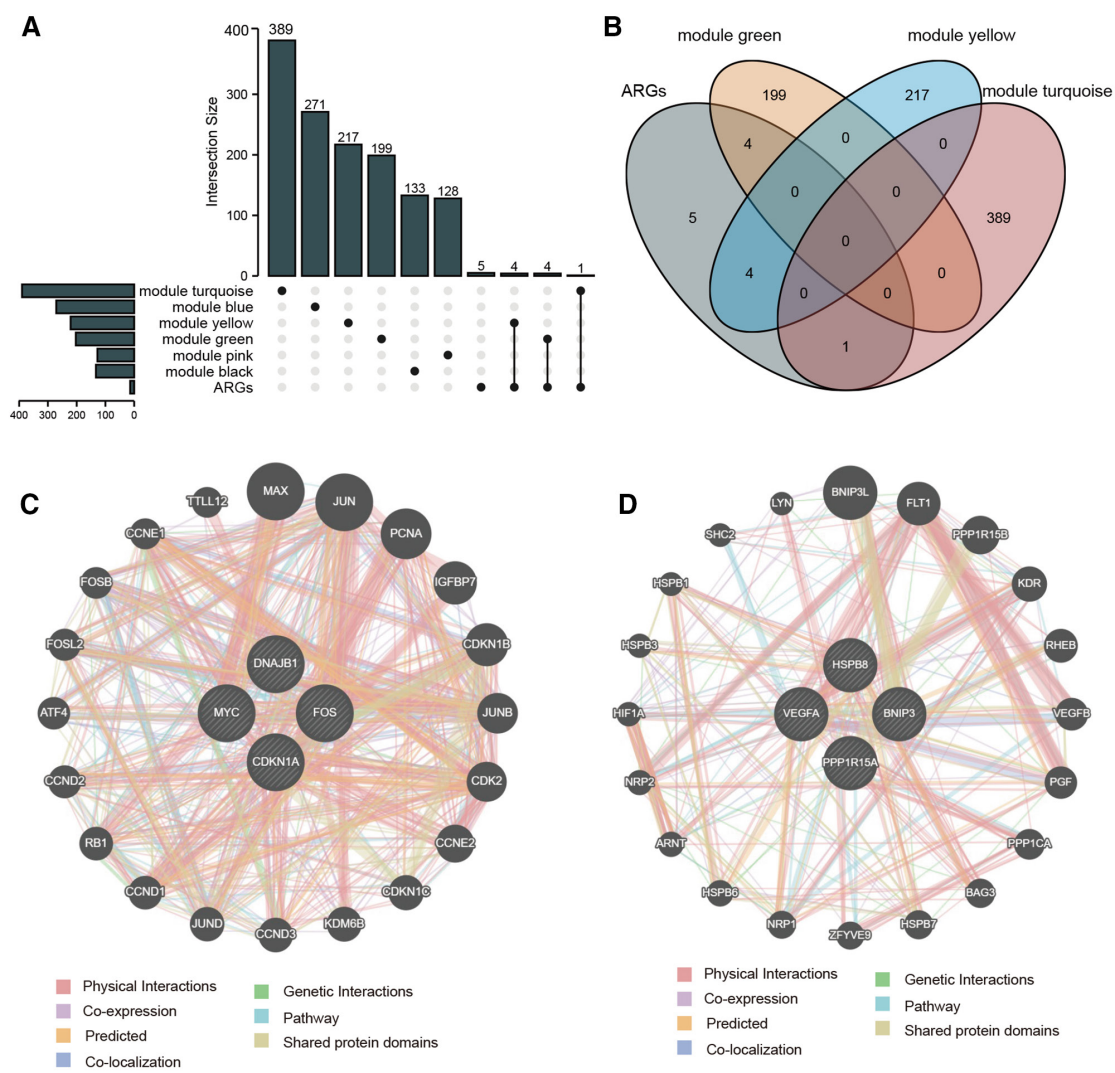
Relationship between the clustering module and differentially expressed ARGs. (A) The UpSet map of clustering modules (MEturquoise, MEpink, MEblue, MEblack, MEyellow, and Megreen) and differentially expressed ARGs. (B) The Venn diagram of clustering modules (MEturquoise, MEyellow, and MEgreen) and differentially expressed ARGs. (C) PPI construction of the shared genes between the MEturquoise module and differentially expressed ARGs. (D) PPI construction of the shared genes between the MEgreen module and differentially expressed ARGs. ARGs = autophagy-related genes; PPI = protein–protein interaction.

Intersecting genes between clustering modules (MEturquoise, MEpink, MEblue, MEblack, MEyellow, and MEgreen) and differentially expressed ARGs were used to plot the UpSet diagram (Fig. 7A) and Venn diagrams (Fig. 7B), respectively. Finally, the results showed shared genes between MEturquoise and differentially expressed ARGs, including *CDKN1A*, *DNAJB1*, *MYC*, *and FOS*, and shared genes between MEgreen and differentially expressed ARGs, including *PPP1R15A*, *VEGFA*, *HSPB8*, and *BNIP3*. This implied the potential autophagy mechanisms involved in MEturquoise and MEgreen modules in KCN. We then separately constructed PPI using the genes from the MEturquoise and MEgreen modules, along with differentially expressed ARGs (Fig. 7C and D).

### 3.4. Identification of molecular subtypes across the KCN samples

**Figure 8.**
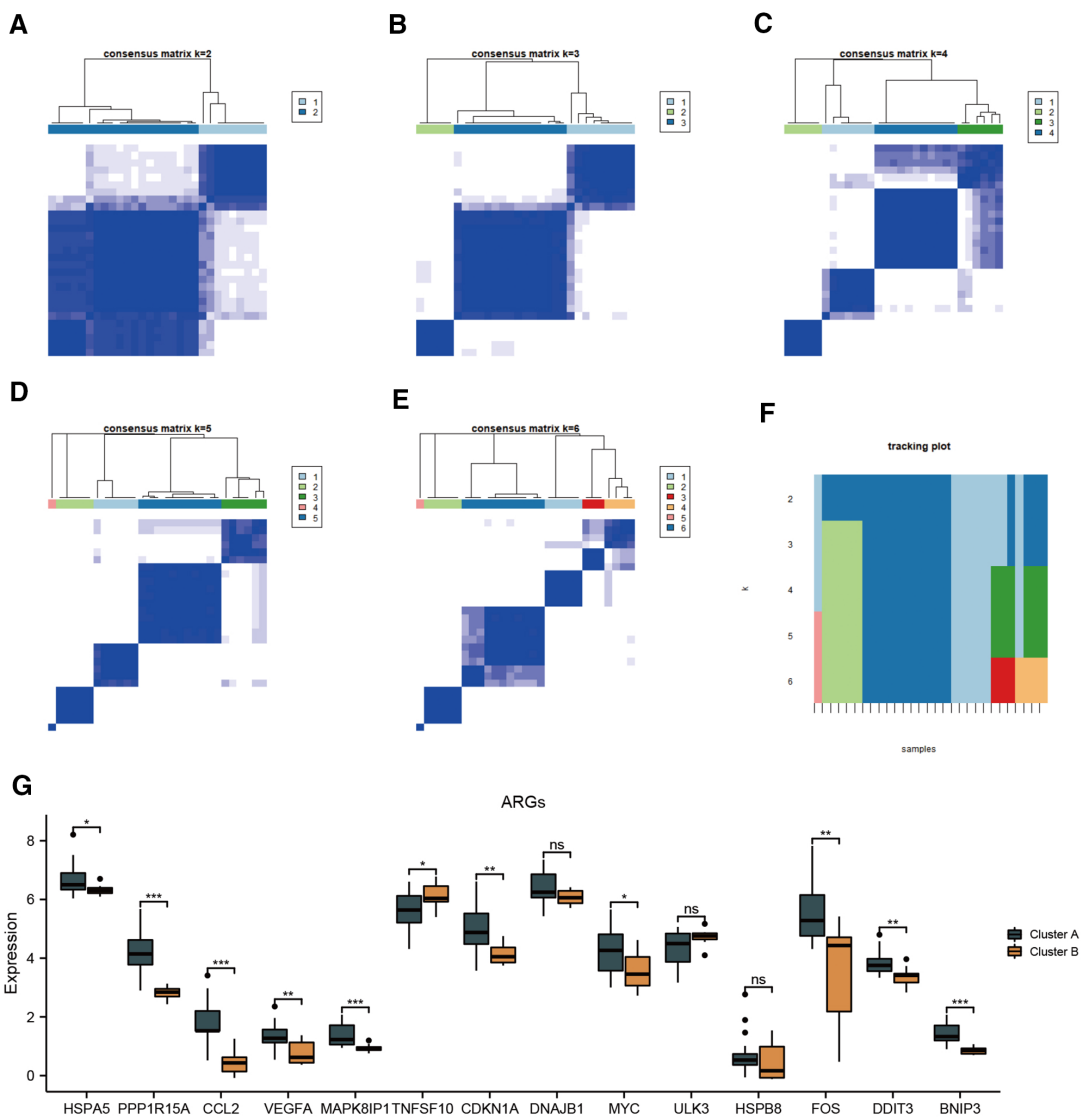
Consensus clustering analysis. (A–E) Consensus matrix plots depicting consensus values on a white-to-blue color scale ordered by consensus clustering (*k* = 2, 3, 4, 5, 6). (F) Tracking plot showing the consensus cluster of items (in the column) at each k (in the row). (G) Box plots showing the difference in mRNA expression of KCN-specific ARGs between clusterA and clusterB (*k* = 2). *P < .05; **P < .01; ***P < .001. ARGs = autophagy-related genes; KCN = keratoconus; mRNA = messenger RNA, ns = no significance.

Consensus clustering analysis of KCN samples clustered different molecular subtypes on the basis of gene expression profiles of KCN-specific ARGs (Fig. 8A–F). The sample clusters exhibited stability and robustness when clustered into 2 subtypes, Therefore, we further analyzed the expression profiles of 14 differentially expressed ARGs between samples when *k* = 2. As shown in Fig. 8G, the expression of KCN-specific ARGs indicated the presence of a heterogeneous subtype. There were 11 significantly downregulated genes in cluster B (*P* < .05), including 9 hub genes (*BNIP3*, *CDKN1A*, *DDIT3*, *FOS*, *HSPA5*, *MAPK8IP1*, *MYC*, *PPP1R15A*, and *VEGFA*).

### 3.5. Immune cell infiltration analysis

**Figure 9.**
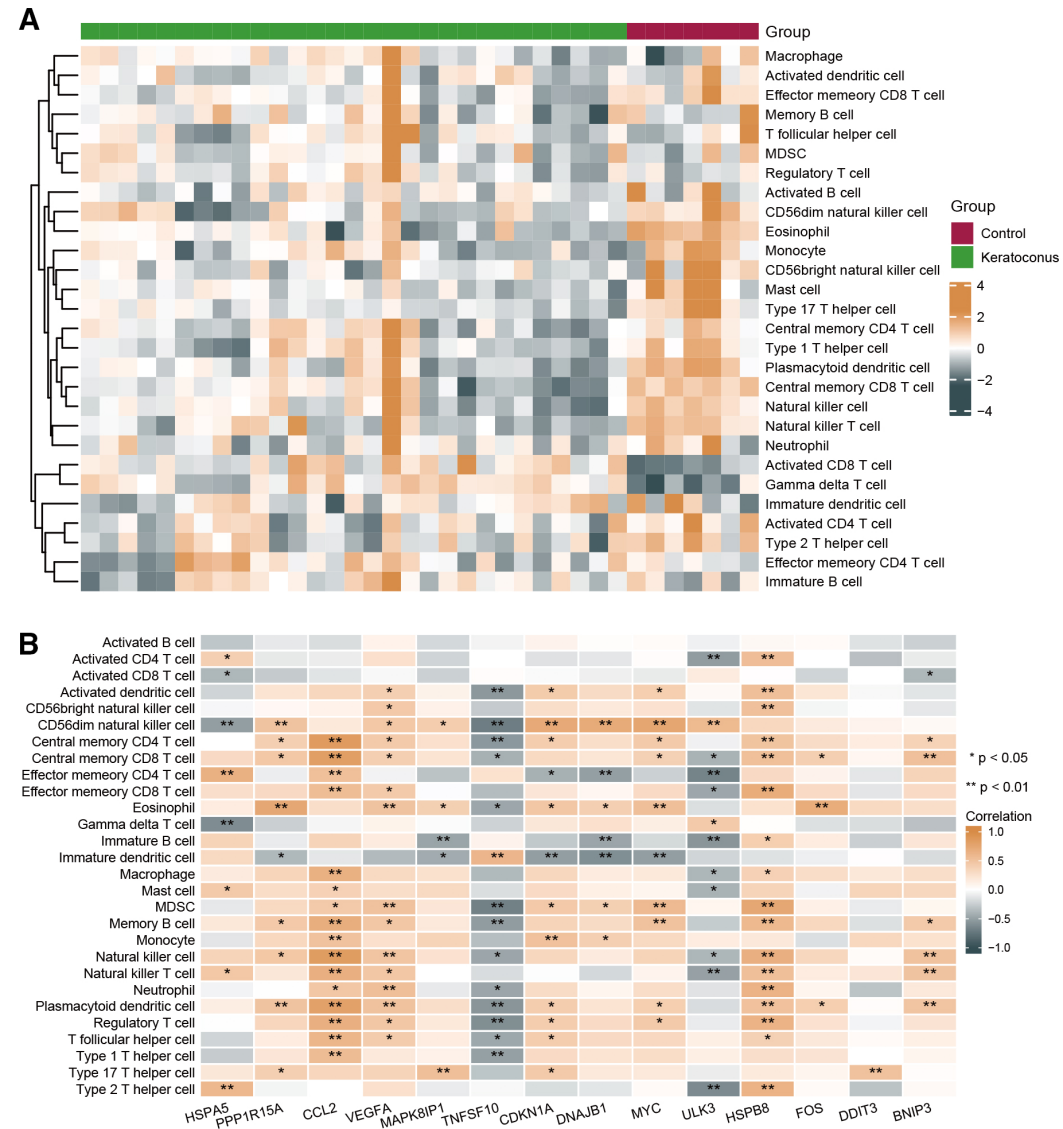
Immune infiltration analysis. (A) The abundance of immune cells in the KCN and control samples based on ssGSEA. (B) Correlation analysis between immune cells and differentially expressed ARGs *P < .05, **P < .01. ARGs = autophagy-related genes; KCN = keratoconus; ssGSEA = single-sample gene set enrichment analysis.

We analyzed multiple different subsets of immune-infiltrating cells across KCN and control samples using the single-sample gene set enrichment analysis method. As shown in Figure 9A, KCN samples exhibited various distribution of immune cell infiltration levels. We further performed a correlation analysis between immune cells and differentially expressed ARGs. Correlation analysis revealed that *CCL2* and *HSPB8* were positively correlated with neutrophils, regulatory T cells, natural killer cells, central memory CD4 T cells, and T follicular helper cells (correlation > 0, *P* < .05); *DDIT3* was positively correlated with type 17 T helper cells (correlation > 0, *P* < .01); *BNIP3* was positively correlated with central memory CD8 T cells, natural killer cells, natural killer T cells, and plasmacytoid dendritic cells (correlation > 0, *P* < .01). In contrast, *TNFSF10* was negatively correlated with type 1 T helper cells, T follicular helper cells, regulatory T cells, neutrophils, memory B cells, and myeloid-derived suppressor cells (correlation < 0; *P* < .05) (Fig. 9B).

### 3.6. Validation of hub genes by RT-qPCR

**Figure 10.**
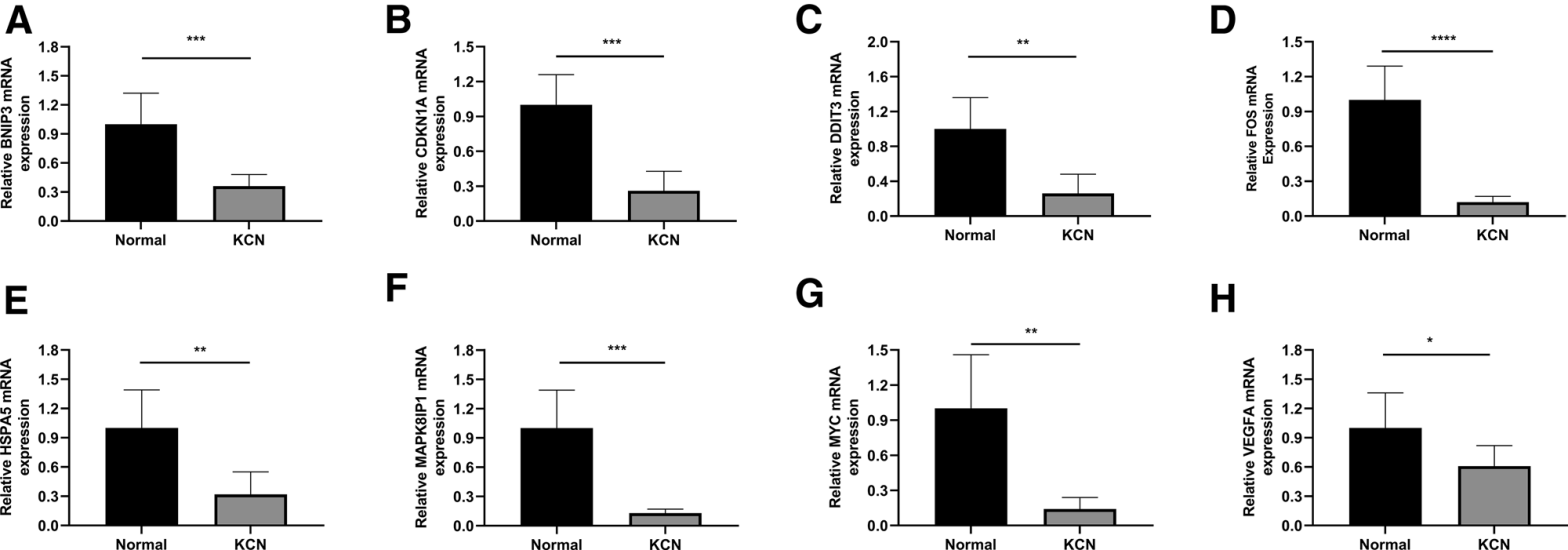
Validation of hub genes by RT-qPCR. The relative mRNA expression of hub genes between patients with keratoconus and healthy controls. The Student *t* test or Mann–Whitney *U*-test were used to compare the differences between the 2 groups. *P < .05, **P < .01, n = 6. mRNA = messenger RNA; RT-qPCR = real-time quantitative polymerase chain reaction.

Nine hub genes were validated using RT-qPCR in corneal samples obtained from patients with KCN and donor controls. The results showed that *BNIP3*, *CDKN1A*, *DDIT3*, *FOS*, *HSPA5*, *MAPK8IP1*, *VEGFA*, and *MYC (P* < .05) were significantly downregulated in the KCN samples (Fig. 10), while *PPP1R15A* expression was too faint to detect.

## 4. Discussion

KCN is a common corneal ectatic disease that results in the deterioration of visual quality, and corneal transplantation is warranted in severe stages. In recent years, high-throughput sequencing has enabled the identification of hub genes and the therapeutic targets of various disease at genome or transcriptional levels.^[24,43]^ Although autophagy has been demonstrated to participate in the pathogenesis of the corneal disease,^[44]^ the mechanism underlying the KCN is largely unknown. The role of autophagy in keratoconus involving multiple mechanisms such as dysregulated gene expression, oxidative stress, and autophagic flux defects. Studies have found significantly elevated expression of the autophagy marker LC3 in both the corneal epithelium and stroma of keratoconus patients^[45]^ and significant abnormalities in the autophagic-lysosomal pathway and also the imbalances between autophagosome formation and lysosomal degradation. The autophagic flux impairment may prevent effective removal of damaged mitochondria and protein aggregates, further exacerbating oxidative damage, resulting in the central cone region of advanced keratoconus.^[15]^ Furthermore, research indicates that autophagy deficiency suppresses lysosomal biogenesis, increasing matrix metalloproteinase activity and accelerating collagen degradation and corneal thinning.^[44]^ However, the signature of ARGs of KCN has not yet been fully studied using high-throughput sequencing data. Therefore, this study aimed to further elucidate the potential role of ARGs in KCN by analyzing high-throughput sequencing data.

In this study, we first screened out 14 hub genes related to autophagy in KCN using NetworkAnalyst and found strong interactions between each gene. We also used a random forest algorithm for the DEGs, and identified 9 overlapping genes (*BNIP*3, *CDKN1A*, *DDIT3*, *FOS*, *HSPA5*, *MAPK8IP1*, *MYC*, *PPP1R15A*, and *VEGFA*). Furthermore, we validated the down-regulated expression of these hub genes using RT-qPCR, which was consistent with the trend observed in the dataset. However, the sample size is limited, future studies with larger cohorts are needed to clarify its role in KCN. The specific molecular mechanisms of these genes in KCN remain unclear. In our study, *BNIP3* and *DDIT3* were downregulated in KCN corneal samples, which is consistent with the results of a previous study on blood samples from patients with KCN.^[46]^ *BNIP3* can regulate the autophagy process in ocular cells, such as retinal ganglion cells^[47]^ and retinal neurons.^[48]^ *DDIT3* is a pro-apoptotic TF that was reported to be markedly upregulated in a human model of corneal epithelial differentiation treated with hyperosmotic saline,^[49]^ and it has also been shown to contribute to retinal ganglion cells somal apoptosis in an ocular hypertensive mouse model of glaucoma.^[50]^ *CDKN1A* was overexpressed in a mouse model of Fuchs endothelial corneal dystrophy and accelerated senescence.^[49]^ *FOS* plays a key role in photodynamic therapy-induced-autophagy in melanoma.^[51]^ *HSPA5* localizes to the lumen of the ER, which is involved in the folding and assembly of proteins in the ER; it is a master regulator of ER homeostasis and is upregulated in Mooren ulcer.^[52]^ *MYC* is TF that plays a central regulatory roles in corneal epithelium maintenance and repair.^[53]^ *MAPK8IP1* was reported to be a key regulator of autophagosome transport in neurons.^[54]^ However, little is known about the role of *MAPK8IP1* in eye diseases. The genes MAPK8IP1, PPP1R15A, and VEGFA are associated with cellular inflammation and apoptosis, and little is known about the role of autophagy mechanism in KCN. The previous studies also identified CDKN1A, HSPA5, MAPK8IP1, PPP1R15A, and VEGFA as hub autophagy related genes, implying potential autophagy mechanism in KCN via the apoptosis or endplasmic reticulum process. Functional enrichment analysis revealed that the ER-nucleus signaling pathway, MAPK signaling pathway, apoptosis, and FoxO signaling pathway are mainly involved. MAPK pathways was involved in controlling the balance between apoptosis and autophagy in various diseases.^[55,56]^ Apoptosis may cause the loosening or the thinning of the corneal tissue structure in KCN.^[57,58]^ Previous studies have also revealed the enrichment of MAPK pathways in corneal stromal cells derived from KCN patients at the transcriptional level,^[59,60]^ suggesting its underlying role in the pathogenesis of KCN. Moreover, FoxO signaling has been reported to be involved in autophagy,^[61]^ although its potential mechanism in KCN has not yet been reported.

KCN is typically been described as a noninflammatory corneal disorder. Nonetheless, accumulating evidence highlights the close interplay between KCN and immune responses.^[18,19,62]^ Recent study on the analysis of single-cell RNA-sequencing and bulk RNA-sequencing data demonstrated the potential roles of immune cells in KCN progression possibly by regulating immunological features and maintaining cell stability.^[63]^ Another study of integrative transcriptomics analysis have also revealed the immunomodulatory patterns are involved in the pathogenesis of KCN.^[5]^ Here, we further analyzed the immune cells in the KCN and control samples. We found a close interaction between immune cell infiltration and the progression of KCN, further demonstrating the participation of the immune system involved in KCN. Moreover, we performed correlation analysis between the hub genes of ARGs and immune cells, revealing the underlying mechanism between autophagy and immune response in KCN; most ARGs were positively correlated with immune cells, whereas *TNFSF10* and *ULK3* were negatively correlated with immunocytes. *HSPA5* and *CCL2* have been reported to be involved in the immune processes of eye disorders^[64,65]^; however, the immune mechanisms underlying KCN warrant further investigation.

We propose a hypothesis: the dysregulation of key genes-including BNIP3-mediated mitophagy impairment, ER stress signaling through HSPA5 and DDIT3, and cellular stress responses modulated by FOS, MYC, and PPP1R15A-disrupts autophagic flux in keratoconus, leading to mitochondrial dysfunction, oxidative stress, and apoptotic activation. Concurrently, altered immune response via MAPK8IP1-driven inflammatory signaling and VEGFA-mediated homeostasis breakdown amplifies corneal inflammation, while CDKN1A-induced cell cycle arrest undermines tissue repair. This autophagy-immune network convergence exacerbates stromal weakening, promoting disease progression through sustained tissue damage and compromised corneal integrity.

This study had several limitations. First, the sample sizes for both bioinformatic analysis and experimental validation were relatively small. The Gene Expression Omnibus datasets included only 7 control samples, and the RT-qPCR validation was performed on only 6 KCN and 6 control samples. This limited sample size may affect statistical power and introduce bias, and thus our findings should be considered preliminary and require validation in larger, independent cohorts. Second, the data were obtained from publicly available datasets, and further experimental studies are needed to confirm the identified genetic signatures and their functional roles in KCN. Third, the mechanistic relationship between autophagy and immune response in KCN remains unclear and necessitates future functional investigations.

## 5. Conclusions

We identified 9 hub genes that related to autophagy (*BNIP3*, *CDKN1A*, *DDIT3*, *FOS*, *HSPA5*, *MAPK8IP1*, *MYC*, *PPP1R15A*, and *VEGFA*), which may contribute to future therapeutic targets in KCN and provides novel theoretical insights into the pathogenesis of KCN. Autophagy mechanisms and immune response were involved in the development of KCN disease. However, due to the limited sample size, further validation of these findings and elucidation of the underlying mechanisms will require larger cohorts and functional experiments.

## Acknowledgments

We are grateful to the investigators who provided the publicly available datasets.

## Author contributions

**Conceptualization:** Naiyang Li.

**Data curation:** Sutong Li, Gang Wang, Jing Chen.

**Formal analysis:** Sutong Li, Gang Wang, Jing Chen.

**Funding acquisition:** Jing Chen.

**Investigation:** Sutong Li.

**Methodology:** Sutong Li.

**Supervision:** Naiyang Li.

**Validation:** Sutong Li, Gang Wang.

**Visualization:** Sutong Li.

**Writing – original draft:** Sutong Li.

**Writing – review & editing:** Naiyang Li.

**Figure:**