# Supplementary material for: Identification and validation of autophagy-related genes in keratoconus and their correlation with immune infiltration
Source: Medicine (Baltimore). 2026 May 29;105(22):e48985. doi: 10.1097/MD.0000000000048985 (PMC13225602; doi:10.1097/MD.0000000000048985)
Supplement: Supplementary file 1 [file medi-105-e48985-s002.docx]

Supplementary Table 1. List of autophagy-related genes

| GeneId | Name | Autophagy Symbol |
| --- | --- | --- |
| [55626](http://www.autophagy.lu/genes/55626) | autophagy/beclin-1 regulator 1 | AMBRA1 |
| [8542](http://www.autophagy.lu/genes/8542) | apolipoprotein L, 1 | APOL1 |
| [405](http://www.autophagy.lu/genes/405) | aryl hydrocarbon receptor nuclear translocator | ARNT |
| [410](http://www.autophagy.lu/genes/410) | arylsulfatase A | ARSA |
| [411](http://www.autophagy.lu/genes/411) | arylsulfatase B | ARSB |
| [468](http://www.autophagy.lu/genes/468) | activating transcription factor 4 (tax-responsive enhancer element B67) | ATF4 |
| [22926](http://www.autophagy.lu/genes/22926) | activating transcription factor 6 | ATF6 |
| [83734](http://www.autophagy.lu/genes/83734) | ATG10 autophagy related 10 homolog (S. cerevisiae) | ATG10 |
| [9140](http://www.autophagy.lu/genes/9140) | ATG12 autophagy related 12 homolog (S. cerevisiae) | ATG12 |
| [55054](http://www.autophagy.lu/genes/55054) | ATG16 autophagy related 16-like 1 (S. cerevisiae) | ATG16L1 |
| [89849](http://www.autophagy.lu/genes/89849) | ATG16 autophagy related 16-like 2 (S. cerevisiae) | ATG16L2 |
| [23130](http://www.autophagy.lu/genes/23130) | ATG2 autophagy related 2 homolog A (S. cerevisiae) | ATG2A |
| [55102](http://www.autophagy.lu/genes/55102) | ATG2 autophagy related 2 homolog B (S. cerevisiae) | ATG2B |
| [64422](http://www.autophagy.lu/genes/64422) | ATG3 autophagy related 3 homolog (S. cerevisiae) | ATG3 |
| [115201](http://www.autophagy.lu/genes/115201) | ATG4 autophagy related 4 homolog A (S. cerevisiae) | ATG4A |
| [23192](http://www.autophagy.lu/genes/23192) | ATG4 autophagy related 4 homolog B (S. cerevisiae) | ATG4B |
| [84938](http://www.autophagy.lu/genes/84938) | ATG4 autophagy related 4 homolog C (S. cerevisiae) | ATG4C |
| [84971](http://www.autophagy.lu/genes/84971) | ATG4 autophagy related 4 homolog D (S. cerevisiae) | ATG4D |
| [9474](http://www.autophagy.lu/genes/9474) | ATG5 autophagy related 5 homolog (S. cerevisiae) | ATG5 |
| [10533](http://www.autophagy.lu/genes/10533) | ATG7 autophagy related 7 homolog (S. cerevisiae) | ATG7 |
| [79065](http://www.autophagy.lu/genes/79065) | ATG9 autophagy related 9 homolog A (S. cerevisiae) | ATG9A |
| [285973](http://www.autophagy.lu/genes/285973) | ATG9 autophagy related 9 homolog B (S. cerevisiae) | ATG9B |
| [471](http://www.autophagy.lu/genes/471) | 5-aminoimidazole-4-carboxamide ribonucleotide formyltransferase/IMP cyclohydrolase | ATIC |
| [573](http://www.autophagy.lu/genes/573) | BCL2-associated athanogene | BAG1 |
| [9531](http://www.autophagy.lu/genes/9531) | BCL2-associated athanogene 3 | BAG3 |
| [578](http://www.autophagy.lu/genes/578) | BCL2-antagonist/killer 1 | BAK1 |
| [581](http://www.autophagy.lu/genes/581) | BCL2-associated X protein | BAX |
| [596](http://www.autophagy.lu/genes/596) | B-cell CLL/lymphoma 2 | BCL2 |
| [598](http://www.autophagy.lu/genes/598) | BCL2-like 1 | BCL2L1 |
| [8678](http://www.autophagy.lu/genes/8678) | beclin 1, autophagy related | BECN1 |
| [637](http://www.autophagy.lu/genes/637) | BH3 interacting domain death agonist | BID |
| [332](http://www.autophagy.lu/genes/332) | baculoviral IAP repeat-containing 5 | BIRC5 |
| [57448](http://www.autophagy.lu/genes/57448) | baculoviral IAP repeat-containing 6 | BIRC6 |
| [662](http://www.autophagy.lu/genes/662) | BCL2/adenovirus E1B 19kDa interacting protein 1 | BNIP1 |
| [664](http://www.autophagy.lu/genes/664) | BCL2/adenovirus E1B 19kDa interacting protein 3 | BNIP3 |
| [665](http://www.autophagy.lu/genes/665) | BCL2/adenovirus E1B 19kDa interacting protein 3-like | BNIP3L |
| [60673](http://www.autophagy.lu/genes/60673) | chromosome 12 open reading frame 44 | C12orf44 |
| [23591](http://www.autophagy.lu/genes/23591) | chromosome 17 open reading frame 88 | C17orf88 |
| [10241](http://www.autophagy.lu/genes/10241) | calcium binding and coiled-coil domain 2 | CALCOCO2 |
| [10645](http://www.autophagy.lu/genes/10645) | calcium/calmodulin-dependent protein kinase kinase 2, beta | CAMKK2 |
| [821](http://www.autophagy.lu/genes/821) | calnexin | CANX |
| [823](http://www.autophagy.lu/genes/823) | calpain 1, (mu/I) large subunit | CAPN1 |
| [11132](http://www.autophagy.lu/genes/11132) | calpain 10 | CAPN10 |
| [824](http://www.autophagy.lu/genes/824) | calpain 2, (m/II) large subunit | CAPN2 |
| [826](http://www.autophagy.lu/genes/826) | calpain, small subunit 1 | CAPNS1 |
| [834](http://www.autophagy.lu/genes/834) | caspase 1, apoptosis-related cysteine peptidase (interleukin 1, beta, convertase) | CASP1 |
| [836](http://www.autophagy.lu/genes/836) | caspase 3, apoptosis-related cysteine peptidase | CASP3 |
| [837](http://www.autophagy.lu/genes/837) | caspase 4, apoptosis-related cysteine peptidase | CASP4 |
| [841](http://www.autophagy.lu/genes/841) | caspase 8, apoptosis-related cysteine peptidase | CASP8 |
| [6347](http://www.autophagy.lu/genes/6347) | chemokine (C-C motif) ligand 2 | CCL2 |
| [729230](http://www.autophagy.lu/genes/729230) | chemokine (C-C motif) receptor 2 | CCR2 |
| [4179](http://www.autophagy.lu/genes/4179) | CD46 molecule, complement regulatory protein | CD46 |
| [1026](http://www.autophagy.lu/genes/1026) | cyclin-dependent kinase inhibitor 1A (p21, Cip1) | CDKN1A |
| [1027](http://www.autophagy.lu/genes/1027) | cyclin-dependent kinase inhibitor 1B (p27, Kip1) | CDKN1B |
| [1029](http://www.autophagy.lu/genes/1029) | cyclin-dependent kinase inhibitor 2A (melanoma, p16, inhibits CDK4) | CDKN2A |
| [8837](http://www.autophagy.lu/genes/8837) | CASP8 and FADD-like apoptosis regulator | CFLAR |
| [25978](http://www.autophagy.lu/genes/25978) | chromatin modifying protein 2B | CHMP2B |
| [128866](http://www.autophagy.lu/genes/128866) | chromatin modifying protein 4B | CHMP4B |
| [1201](http://www.autophagy.lu/genes/1201) | ceroid-lipofuscinosis, neuronal 3 | CLN3 |
| [1508](http://www.autophagy.lu/genes/1508) | cathepsin B | CTSB |
| [1509](http://www.autophagy.lu/genes/1509) | cathepsin D | CTSD |
| [1514](http://www.autophagy.lu/genes/1514) | cathepsin L1 | CTSL1 |
| [6376](http://www.autophagy.lu/genes/6376) | chemokine (C-X3-C motif) ligand 1 | CX3CL1 |
| [7852](http://www.autophagy.lu/genes/7852) | chemokine (C-X-C motif) receptor 4 | CXCR4 |
| [1612](http://www.autophagy.lu/genes/1612) | death-associated protein kinase 1 | DAPK1 |
| [23604](http://www.autophagy.lu/genes/23604) | death-associated protein kinase 2 | DAPK2 |
| [1649](http://www.autophagy.lu/genes/1649) | DNA-damage-inducible transcript 3 | DDIT3 |
| [9077](http://www.autophagy.lu/genes/9077) | DIRAS family, GTP-binding RAS-like 3 | DIRAS3 |
| [10395](http://www.autophagy.lu/genes/10395) | deleted in liver cancer 1 | DLC1 |
| [3337](http://www.autophagy.lu/genes/3337) | DnaJ (Hsp40) homolog, subfamily B, member 1 | DNAJB1 |
| [4189](http://www.autophagy.lu/genes/4189) | DnaJ (Hsp40) homolog, subfamily B, member 9 | DNAJB9 |
| [55332](http://www.autophagy.lu/genes/55332) | DNA-damage regulated autophagy modulator 1 | DRAM1 |
| [9695](http://www.autophagy.lu/genes/9695) | ER degradation enhancer, mannosidase alpha-like 1 | EDEM1 |
| [1938](http://www.autophagy.lu/genes/1938) | eukaryotic translation elongation factor 2 | EEF2 |
| [29904](http://www.autophagy.lu/genes/29904) | eukaryotic elongation factor-2 kinase | EEF2K |
| [1956](http://www.autophagy.lu/genes/1956) | epidermal growth factor receptor (erythroblastic leukemia viral (v-erb-b) oncogene homolog, avian) | EGFR |
| [5610](http://www.autophagy.lu/genes/5610) | eukaryotic translation initiation factor 2-alpha kinase 2 | EIF2AK2 |
| [9451](http://www.autophagy.lu/genes/9451) | eukaryotic translation initiation factor 2-alpha kinase 3 | EIF2AK3 |
| [1965](http://www.autophagy.lu/genes/1965) | eukaryotic translation initiation factor 2, subunit 1 alpha, 35kDa | EIF2S1 |
| [1978](http://www.autophagy.lu/genes/1978) | eukaryotic translation initiation factor 4E binding protein 1 | EIF4EBP1 |
| [1981](http://www.autophagy.lu/genes/1981) | eukaryotic translation initiation factor 4 gamma, 1 | EIF4G1 |
| [2064](http://www.autophagy.lu/genes/2064) | v-erb-b2 erythroblastic leukemia viral oncogene homolog 2, neuro/glioblastoma derived oncogene homolog (avian) | ERBB2 |
| [2081](http://www.autophagy.lu/genes/2081) | endoplasmic reticulum to nucleus signaling 1 | ERN1 |
| [30001](http://www.autophagy.lu/genes/30001) | ERO1-like (S. cerevisiae) | ERO1L |
| [8772](http://www.autophagy.lu/genes/8772) | Fas (TNFRSF6)-associated via death domain | FADD |
| [55578](http://www.autophagy.lu/genes/55578) | family with sequence similarity 48, member A | FAM48A |
| [355](http://www.autophagy.lu/genes/355) | Fas (TNF receptor superfamily, member 6) | FAS |
| [2280](http://www.autophagy.lu/genes/2280) | FK506 binding protein 1A, 12kDa | FKBP1A |
| [2281](http://www.autophagy.lu/genes/2281) | FK506 binding protein 1B, 12.6 kDa | FKBP1B |
| [2353](http://www.autophagy.lu/genes/2353) | FBJ murine osteosarcoma viral oncogene homolog | FOS |
| [2308](http://www.autophagy.lu/genes/2308) | forkhead box O1 | FOXO1 |
| [2309](http://www.autophagy.lu/genes/2309) | forkhead box O3 | FOXO3 |
| [2548](http://www.autophagy.lu/genes/2548) | glucosidase, alpha; acid | GAA |
| [11337](http://www.autophagy.lu/genes/11337) | GABA(A) receptor-associated protein | GABARAP |
| [23710](http://www.autophagy.lu/genes/23710) | GABA(A) receptor-associated protein like 1 | GABARAPL1 |
| [11345](http://www.autophagy.lu/genes/11345) | GABA(A) receptor-associated protein-like 2 | GABARAPL2 |
| [2597](http://www.autophagy.lu/genes/2597) | glyceraldehyde-3-phosphate dehydrogenase | GAPDH |
| [2773](http://www.autophagy.lu/genes/2773) | guanine nucleotide binding protein (G protein), alpha inhibiting activity polypeptide 3 | GNAI3 |
| [10399](http://www.autophagy.lu/genes/10399) | guanine nucleotide binding protein (G protein), beta polypeptide 2-like 1 | GNB2L1 |
| [57120](http://www.autophagy.lu/genes/57120) | golgi-associated PDZ and coiled-coil motif containing | GOPC |
| [2894](http://www.autophagy.lu/genes/2894) | glutamate receptor, ionotropic, delta 1 | GRID1 |
| [2895](http://www.autophagy.lu/genes/2895) | glutamate receptor, ionotropic, delta 2 | GRID2 |
| [3065](http://www.autophagy.lu/genes/3065) | histone deacetylase 1 | HDAC1 |
| [10013](http://www.autophagy.lu/genes/10013) | histone deacetylase 6 | HDAC6 |
| [9146](http://www.autophagy.lu/genes/9146) | hepatocyte growth factor-regulated tyrosine kinase substrate | HGS |
| [3091](http://www.autophagy.lu/genes/3091) | hypoxia inducible factor 1, alpha subunit (basic helix-loop-helix transcription factor) | HIF1A |
| [3326](http://www.autophagy.lu/genes/3326) | heat shock protein 90kDa alpha (cytosolic), class B member 1 | HSP90AB1 |
| [3309](http://www.autophagy.lu/genes/3309) | heat shock 70kDa protein 5 (glucose-regulated protein, 78kDa) | HSPA5 |
| [3312](http://www.autophagy.lu/genes/3312) | heat shock 70kDa protein 8 | HSPA8 |
| [26353](http://www.autophagy.lu/genes/26353) | heat shock 22kDa protein 8 | HSPB8 |
| [3458](http://www.autophagy.lu/genes/3458) | interferon, gamma | IFNG |
| [3551](http://www.autophagy.lu/genes/3551) | inhibitor of kappa light polypeptide gene enhancer in B-cells, kinase beta | IKBKB |
| [9641](http://www.autophagy.lu/genes/9641) | inhibitor of kappa light polypeptide gene enhancer in B-cells, kinase epsilon | IKBKE |
| [11009](http://www.autophagy.lu/genes/11009) | interleukin 24 | IL24 |
| [345611](http://www.autophagy.lu/genes/345611) | immunity-related GTPase family, M | IRGM |
| [3675](http://www.autophagy.lu/genes/3675) | integrin, alpha 3 (antigen CD49C, alpha 3 subunit of VLA-3 receptor) | ITGA3 |
| [3655](http://www.autophagy.lu/genes/3655) | integrin, alpha 6 | ITGA6 |
| [3688](http://www.autophagy.lu/genes/3688) | integrin, beta 1 (fibronectin receptor, beta polypeptide, antigen CD29 includes MDF2, MSK12) | ITGB1 |
| [3691](http://www.autophagy.lu/genes/3691) | integrin, beta 4 | ITGB4 |
| [3708](http://www.autophagy.lu/genes/3708) | inositol 1,4,5-triphosphate receptor, type 1 | ITPR1 |
| [2548](http://www.autophagy.lu/genes/2548) | glucosidase, alpha; acid | GAA |
| [11337](http://www.autophagy.lu/genes/11337) | GABA(A) receptor-associated protein | GABARAP |
| [23710](http://www.autophagy.lu/genes/23710) | GABA(A) receptor-associated protein like 1 | GABARAPL1 |
| [11345](http://www.autophagy.lu/genes/11345) | GABA(A) receptor-associated protein-like 2 | GABARAPL2 |
| [2597](http://www.autophagy.lu/genes/2597) | glyceraldehyde-3-phosphate dehydrogenase | GAPDH |
| [2773](http://www.autophagy.lu/genes/2773) | guanine nucleotide binding protein (G protein), alpha inhibiting activity polypeptide 3 | GNAI3 |
| [10399](http://www.autophagy.lu/genes/10399) | guanine nucleotide binding protein (G protein), beta polypeptide 2-like 1 | GNB2L1 |
| [57120](http://www.autophagy.lu/genes/57120) | golgi-associated PDZ and coiled-coil motif containing | GOPC |
| [2894](http://www.autophagy.lu/genes/2894) | glutamate receptor, ionotropic, delta 1 | GRID1 |
| [2895](http://www.autophagy.lu/genes/2895) | glutamate receptor, ionotropic, delta 2 | GRID2 |
| [9711](http://www.autophagy.lu/genes/9711) | KIAA0226 | KIAA0226 |
| [9776](http://www.autophagy.lu/genes/9776) | KIAA0652 | KIAA0652 |
| [22863](http://www.autophagy.lu/genes/22863) | KIAA0831 | KIAA0831 |
| [3799](http://www.autophagy.lu/genes/3799) | kinesin family member 5B | KIF5B |
| [54800](http://www.autophagy.lu/genes/54800) | kelch-like 24 (Drosophila) | KLHL24 |
| [3916](http://www.autophagy.lu/genes/3916) | lysosomal-associated membrane protein 1 | LAMP1 |
| [3920](http://www.autophagy.lu/genes/3920) | lysosomal-associated membrane protein 2 | LAMP2 |
| [84557](http://www.autophagy.lu/genes/84557) | microtubule-associated protein 1 light chain 3 alpha | MAP1LC3A |
| [81631](http://www.autophagy.lu/genes/81631) | microtubule-associated protein 1 light chain 3 beta | MAP1LC3B |
| [440738](http://www.autophagy.lu/genes/440738) | microtubule-associated protein 1 light chain 3 gamma | MAP1LC3C |
| [5609](http://www.autophagy.lu/genes/5609) | mitogen-activated protein kinase kinase 7 | MAP2K7 |
| [5594](http://www.autophagy.lu/genes/5594) | mitogen-activated protein kinase 1 | MAPK1 |
| [5595](http://www.autophagy.lu/genes/5595) | mitogen-activated protein kinase 3 | MAPK3 |
| [5599](http://www.autophagy.lu/genes/5599) | mitogen-activated protein kinase 8 | MAPK8 |
| [9479](http://www.autophagy.lu/genes/9479) | mitogen-activated protein kinase 8 interacting protein 1 | MAPK8IP1 |
| [5601](http://www.autophagy.lu/genes/5601) | mitogen-activated protein kinase 9 | MAPK9 |
| [51360](http://www.autophagy.lu/genes/51360) | membrane-bound transcription factor peptidase, site 2 | MBTPS2 |
| [64223](http://www.autophagy.lu/genes/64223) | MTOR associated protein, LST8 homolog (S. cerevisiae) | MLST8 |
| [64419](http://www.autophagy.lu/genes/64419) | myotubularin related protein 14 | MTMR14 |
| [2475](http://www.autophagy.lu/genes/2475) | mechanistic target of rapamycin (serine/threonine kinase) | MTOR |
| [4609](http://www.autophagy.lu/genes/4609) | v-myc myelocytomatosis viral oncogene homolog (avian) | MYC |
| [92345](http://www.autophagy.lu/genes/92345) | nuclear assembly factor 1 homolog (S. cerevisiae) | NAF1 |
| [10135](http://www.autophagy.lu/genes/10135) | nicotinamide phosphoribosyltransferase | NAMPT |
| [4077](http://www.autophagy.lu/genes/4077) | neighbor of BRCA1 gene 1 | NBR1 |
| [10787](http://www.autophagy.lu/genes/10787) | NCK-associated protein 1 | NCKAP1 |
| [4780](http://www.autophagy.lu/genes/4780) | nuclear factor (erythroid-derived 2)-like 2 | NFE2L2 |
| [4790](http://www.autophagy.lu/genes/4790) | nuclear factor of kappa light polypeptide gene enhancer in B-cells 1 | NFKB1 |
| [159296](http://www.autophagy.lu/genes/159296) | NK2 transcription factor related, locus 3 (Drosophila) | NKX2-3 |
| [58484](http://www.autophagy.lu/genes/58484) | NLR family, CARD domain containing 4 | NLRC4 |
| [4864](http://www.autophagy.lu/genes/4864) | Niemann-Pick disease, type C1 | NPC1 |
| [3084](http://www.autophagy.lu/genes/3084) | neuregulin 1 | NRG1 |
| [9542](http://www.autophagy.lu/genes/9542) | neuregulin 2 | NRG2 |
| [10718](http://www.autophagy.lu/genes/10718) | neuregulin 3 | NRG3 |
| [5034](http://www.autophagy.lu/genes/5034) | prolyl 4-hydroxylase, beta polypeptide | P4HB |
| [5071](http://www.autophagy.lu/genes/5071) | Parkinson disease (autosomal recessive, juvenile) 2, parkin | PARK2 |
| [142](http://www.autophagy.lu/genes/142) | poly (ADP-ribose) polymerase 1 | PARP1 |
| [8682](http://www.autophagy.lu/genes/8682) | phosphoprotein enriched in astrocytes 15 | PEA15 |
| [27043](http://www.autophagy.lu/genes/27043) | proline, glutamate and leucine rich protein 1 | PELP1 |
| [5195](http://www.autophagy.lu/genes/5195) | peroxisomal biogenesis factor 14 | PEX14 |
| [8504](http://www.autophagy.lu/genes/8504) | peroxisomal biogenesis factor 3 | PEX3 |
| [5289](http://www.autophagy.lu/genes/5289) | phosphoinositide-3-kinase, class 3 | PIK3C3 |
| [30849](http://www.autophagy.lu/genes/30849) | phosphoinositide-3-kinase, regulatory subunit 4 | PIK3R4 |
| [65018](http://www.autophagy.lu/genes/65018) | PTEN induced putative kinase 1 | PINK1 |
| [23645](http://www.autophagy.lu/genes/23645) | protein phosphatase 1, regulatory (inhibitor) subunit 15A | PPP1R15A |
| [5564](http://www.autophagy.lu/genes/5564) | protein kinase, AMP-activated, beta 1 non-catalytic subunit | PRKAB1 |
| [5573](http://www.autophagy.lu/genes/5573) | protein kinase, cAMP-dependent, regulatory, type I, alpha (tissue specific extinguisher 1) | PRKAR1A |
| [5580](http://www.autophagy.lu/genes/5580) | protein kinase C, delta | PRKCD |
| [5588](http://www.autophagy.lu/genes/5588) | protein kinase C, theta | PRKCQ |
| [5728](http://www.autophagy.lu/genes/5728) | phosphatase and tensin homolog | PTEN |
| [5753](http://www.autophagy.lu/genes/5753) | PTK6 protein tyrosine kinase 6 | PTK6 |
| [8766](http://www.autophagy.lu/genes/8766) | RAB11A, member RAS oncogene family | RAB11A |
| [5861](http://www.autophagy.lu/genes/5861) | RAB1A, member RAS oncogene family | RAB1A |
| [53917](http://www.autophagy.lu/genes/53917) | RAB24, member RAS oncogene family | RAB24 |
| [83452](http://www.autophagy.lu/genes/83452) | RAB33B, member RAS oncogene family | RAB33B |
| [5868](http://www.autophagy.lu/genes/5868) | RAB5A, member RAS oncogene family | RAB5A |
| [7879](http://www.autophagy.lu/genes/7879) | RAB7A, member RAS oncogene family | RAB7A |
| [5879](http://www.autophagy.lu/genes/5879) | ras-related C3 botulinum toxin substrate 1 (rho family, small GTP binding protein Rac1) | RAC1 |
| [5894](http://www.autophagy.lu/genes/5894) | v-raf-1 murine leukemia viral oncogene homolog 1 | RAF1 |
| [5925](http://www.autophagy.lu/genes/5925) | retinoblastoma 1 | RB1 |
| [9821](http://www.autophagy.lu/genes/9821) | RB1-inducible coiled-coil 1 | RB1CC1 |
| [5970](http://www.autophagy.lu/genes/5970) | v-rel reticuloendotheliosis viral oncogene homolog A (avian) | RELA |
| [10287](http://www.autophagy.lu/genes/10287) | regulator of G-protein signaling 19 | RGS19 |
| [6009](http://www.autophagy.lu/genes/6009) | Ras homolog enriched in brain | RHEB |
| [6198](http://www.autophagy.lu/genes/6198) | ribosomal protein S6 kinase, 70kDa, polypeptide 1 | RPS6KB1 |
| [57521](http://www.autophagy.lu/genes/57521) | regulatory associated protein of MTOR, complex 1 | RPTOR |
| [56681](http://www.autophagy.lu/genes/56681) | SAR1 homolog A (S. cerevisiae) | SAR1A |
| [5265](http://www.autophagy.lu/genes/5265) | serpin peptidase inhibitor, clade A (alpha-1 antiproteinase, antitrypsin), member 1 | SERPINA1 |
| [83667](http://www.autophagy.lu/genes/83667) | sestrin 2 | SESN2 |
| [51100](http://www.autophagy.lu/genes/51100) | SH3-domain GRB2-like endophilin B1 | SH3GLB1 |
| [23411](http://www.autophagy.lu/genes/23411) | sirtuin (silent mating type information regulation 2 homolog) 1 (S. cerevisiae) | SIRT1 |
| [22933](http://www.autophagy.lu/genes/22933) | sirtuin (silent mating type information regulation 2 homolog) 2 (S. cerevisiae) | SIRT2 |
| [8877](http://www.autophagy.lu/genes/8877) | sphingosine kinase 1 | SPHK1 |
| [83985](http://www.autophagy.lu/genes/83985) | spinster homolog 1 (Drosophila) | SPNS1 |
| [8878](http://www.autophagy.lu/genes/8878) | sequestosome 1 | SQSTM1 |
| [6767](http://www.autophagy.lu/genes/6767) | suppression of tumorigenicity 13 (colon carcinoma) (Hsp70 interacting protein) | ST13 |
| [6794](http://www.autophagy.lu/genes/6794) | serine/threonine kinase 11 | STK11 |
| [29110](http://www.autophagy.lu/genes/29110) | TANK-binding kinase 1 | TBK1 |
| [10548](http://www.autophagy.lu/genes/10548) | transmembrane 9 superfamily member 1 | TM9SF1 |
| [81671](http://www.autophagy.lu/genes/81671) | transmembrane protein 49 | TMEM49 |
| [157753](http://www.autophagy.lu/genes/157753) | transmembrane protein 74 | TMEM74 |
| [8743](http://www.autophagy.lu/genes/8743) | tumor necrosis factor (ligand) superfamily, member 10 | TNFSF10 |
| [7157](http://www.autophagy.lu/genes/7157) | tumor protein p53 | TP53 |
| [58476](http://www.autophagy.lu/genes/58476) | tumor protein p53 inducible nuclear protein 2 | TP53INP2 |
| [8626](http://www.autophagy.lu/genes/8626) | tumor protein p63 | TP63 |
| [7161](http://www.autophagy.lu/genes/7161) | tumor protein p73 | TP73 |
| [7248](http://www.autophagy.lu/genes/7248) | tuberous sclerosis 1 | TSC1 |
| [7249](http://www.autophagy.lu/genes/7249) | tuberous sclerosis 2 | TSC2 |
| [286319](http://www.autophagy.lu/genes/286319) | tumor suppressor candidate 1 | TUSC1 |
| [8408](http://www.autophagy.lu/genes/8408) | unc-51-like kinase 1 (C. elegans) | ULK1 |
| [9706](http://www.autophagy.lu/genes/9706) | unc-51-like kinase 2 (C. elegans) | ULK2 |
| [25989](http://www.autophagy.lu/genes/25989) | unc-51-like kinase 3 (C. elegans) | ULK3 |
| [9100](http://www.autophagy.lu/genes/9100) | ubiquitin specific peptidase 10 | USP10 |
| [7405](http://www.autophagy.lu/genes/7405) | UV radiation resistance associated gene | UVRAG |
| [9341](http://www.autophagy.lu/genes/9341) | vesicle-associated membrane protein 3 (cellubrevin) | VAMP3 |
| [6845](http://www.autophagy.lu/genes/6845) | vesicle-associated membrane protein 7 | VAMP7 |
| [7422](http://www.autophagy.lu/genes/7422) | vascular endothelial growth factor A | VEGFA |
| [23001](http://www.autophagy.lu/genes/23001) | WD repeat and FYVE domain containing 3 | WDFY3 |
| [11152](http://www.autophagy.lu/genes/11152) | WD repeat domain 45 | WDR45 |
| [56270](http://www.autophagy.lu/genes/56270) | WDR45-like | WDR45L |
| [55062](http://www.autophagy.lu/genes/55062) | WD repeat domain, phosphoinositide interacting 1 | WIPI1 |
| [26100](http://www.autophagy.lu/genes/26100) | WD repeat domain, phosphoinositide interacting 2 | WIPI2 |
| [53349](http://www.autophagy.lu/genes/53349) | zinc finger, FYVE domain containing 1 | ZFYVE1 |
