# Supplementary material for: Identification and validation of autophagy-related genes in keratoconus and their correlation with immune infiltration
Source: Medicine (Baltimore). 2026 May 29;105(22):e48985. doi: 10.1097/MD.0000000000048985 (PMC13225602; doi:10.1097/MD.0000000000048985)
Supplement: Supplementary file 2 [file medi-105-e48985-s003.docx]

Supplementary Table 2. RT-qPCR primer sequences used in this study

| Name | Primer sequences (5′–3′) |  |
| --- | --- | --- |
| HSPA5-F | CATCACGCCGTCCTATGTCG | |
| HSPA5-R | CGTCAAAGACCGTGTTCTCG | |
| GAPDH-F | CAAGAGCACAAGAGGAAGAGAG | |
| GAPDH-R | CTACATGGCAACTGTGAGGAG | |
| PPP1R15A-F | GACCCCTCCAACTCTCCTTC | |
| PPP1R15A-R | CTTCCTCAGCCTCAGCATTC | |
| VEGFA-F | ACAGAACGATCGATACAGAA | |
| VEGFA-R | AAAGATCATGCCAGAGTCTC | |
| MAPK8IP1-F | ATCGCTTCGCCTCCCAATTT | |
| MAPK8IP1-R | ATCTCCGAGAGGTCTTCATCC | |
| CDKN1A-F | CGATGGAACTTCGACTTTGTCA | |
| CDKN1A-R | GCACAAGGGTACAAGACAGTG | |
| MYC-F | CAGCTGCTTAGACGCTGGATT | |
| MYC-R | GTAGAAATACGGCTGCACCGA | |
| FOS-F | CTGGCGTTGTGAAGACCAT | |
| FOS-R | TCCCTTCGGATTCTCCTTTT | |
| DDIT3-F | GGAAACAGAGTGGTCATTCCC | |
| DDIT3-R | CTGCTTGAGCCGTTCATTCTC | |
| BNIP3-F | TTGGATGCACAACATGAATCAGG | |
| BNIP3-R | TCTTCTGACTGAGAGCTATGGTC | |
