# Supplementary material for: Identification and validation of autophagy-related genes in keratoconus and their correlation with immune infiltration
Source: Medicine (Baltimore). 2026 May 29;105(22):e48985. doi: 10.1097/MD.0000000000048985 (PMC13225602; doi:10.1097/MD.0000000000048985)
Supplement: Supplementary file 3 [file medi-105-e48985-s004.docx]

Supplementary Table 3. GO functional analysis

| ONTOLOGY | ID | Description | Gene  Ratio | BgRatio | p-value | p.adjust |
| --- | --- | --- | --- | --- | --- | --- |
| BP | GO:0036499 | PERK-mediated unfolded protein response | 4/14 | 21/18670 | 1.17518E-09 | 9.67851E-07 |
| BP | GO:0006986 | response to unfolded protein | 6/14 | 176/18670 | 1.81756E-09 | 9.67851E-07 |
| BP | GO:0035966 | response to topologically incorrect protein | 6/14 | 199/18670 | 3.80363E-09 | 1.35029E-06 |
| BP | GO:0006984 | ER-nucleus signaling pathway | 4/14 | 46/18670 | 3.17004E-08 | 8.44022E-06 |
| BP | GO:0034620 | cellular response to unfolded protein | 5/14 | 140/18670 | 4.18445E-08 | 8.91288E-06 |
| BP | GO:0035967 | cellular response to topologically incorrect protein | 5/14 | 161/18670 | 8.42506E-08 | 1.49545E-05 |
| BP | GO:0009266 | response to temperature stimulus | 5/14 | 243/18670 | 6.52096E-07 | 9.92118E-05 |
| BP | GO:0097193 | intrinsic apoptotic signaling pathway | 5/14 | 289/18670 | 1.53301E-06 | 0.000170187 |
| BP | GO:0030968 | endoplasmic reticulum unfolded protein response | 4/14 | 121/18670 | 1.598E-06 | 0.000170187 |
| BP | GO:0043618 | regulation of transcription from RNA polymerase II promoter in response to stress | 4/14 | 121/18670 | 1.598E-06 | 0.000170187 |
| CC | GO:0090575 | RNA polymerase II transcription factor complex | 2/14 | 163/19717 | 0.005790764 | 0.05884273 |
| CC | GO:0005741 | mitochondrial outer membrane | 2/14 | 178/19717 | 0.00686734 | 0.05884273 |
| CC | GO:0000164 | protein phosphatase type 1 complex | 1/14 | 10/19717 | 0.007079438 | 0.05884273 |
| CC | GO:0034663 | endoplasmic reticulum chaperone complex | 1/14 | 11/19717 | 0.007784816 | 0.05884273 |
| CC | GO:0061827 | sperm head | 1/14 | 11/19717 | 0.007784816 | 0.05884273 |
| CC | GO:0031968 | organelle outer membrane | 2/14 | 201/19717 | 0.008681157 | 0.05884273 |
| CC | GO:0044798 | nuclear transcription factor complex | 2/14 | 201/19717 | 0.008681157 | 0.05884273 |
| CC | GO:0032993 | protein-DNA complex | 2/14 | 202/19717 | 0.00876442 | 0.05884273 |
| CC | GO:0019867 | outer membrane | 2/14 | 203/19717 | 0.008848046 | 0.05884273 |
| CC | GO:0044292 | dendrite terminus | 1/14 | 13/19717 | 0.009194177 | 0.05884273 |
| MF | GO:0005125 | cytokine activity | 3/14 | 220/17697 | 0.000623423 | 0.020516224 |
| MF | GO:0001047 | core promoter binding | 2/14 | 55/17697 | 0.000842594 | 0.020516224 |
| MF | GO:0004860 | protein kinase inhibitor activity | 2/14 | 63/17697 | 0.00110414 | 0.020516224 |
| MF | GO:0019210 | kinase inhibitor activity | 2/14 | 67/17697 | 0.001247745 | 0.020516224 |
| MF | GO:0005126 | cytokine receptor binding | 3/14 | 286/17697 | 0.001332222 | 0.020516224 |
| MF | GO:0033613 | activating transcription factor binding | 2/14 | 85/17697 | 0.001998362 | 0.025645641 |
| MF | GO:0051087 | chaperone binding | 2/14 | 102/17697 | 0.002861299 | 0.031474289 |
| MF | GO:0031072 | heat shock protein binding | 2/14 | 119/17697 | 0.003870235 | 0.035946896 |
| MF | GO:0001228 | DNA-binding transcription activator activity, RNA polymerase II-specific | 3/14 | 439/17697 | 0.004501263 | 0.035946896 |
| MF | GO:0051082 | unfolded protein binding | 2/14 | 131/17697 | 0.004668428 | 0.035946896 |
