# Supplementary material for: Identification and validation of autophagy-related genes in keratoconus and their correlation with immune infiltration
Source: Medicine (Baltimore). 2026 May 29;105(22):e48985. doi: 10.1097/MD.0000000000048985 (PMC13225602; doi:10.1097/MD.0000000000048985)
Supplement: Supplementary file 4 [file medi-105-e48985-s005.docx]

Supplementary Table 4. KEGG functional analysis

| ID | Description | GeneRatio | BgRatio | pvalue | p.adjust |
| --- | --- | --- | --- | --- | --- |
| hsa05219 | Bladder cancer | 3/12 | 41/8076 | 0.0000 | 0.0021 |
| hsa04010 | MAPK signaling pathway | 5/12 | 294/8076 | 0.0000 | 0.0021 |
| hsa04141 | Protein processing in endoplasmic reticulum | 4/12 | 171/8076 | 0.0001 | 0.0030 |
| hsa05167 | Kaposi sarcoma-associated herpesvirus infection | 4/12 | 193/8076 | 0.0001 | 0.0036 |
| hsa05210 | Colorectal cancer | 3/12 | 86/8076 | 0.0002 | 0.0043 |
| hsa05163 | Human cytomegalovirus infection | 4/12 | 225/8076 | 0.0002 | 0.0043 |
| hsa05323 | Rheumatoid arthritis | 3/12 | 93/8076 | 0.0003 | 0.0046 |
| hsa04068 | FoxO signaling pathway | 3/12 | 131/8076 | 0.0008 | 0.0105 |
| hsa04210 | Apoptosis | 3/12 | 136/8076 | 0.0009 | 0.0105 |
| hsa05418 | Fluid shear stress and atherosclerosis | 3/12 | 139/8076 | 0.0010 | 0.0105 |
