# Supplementary material for: Identification and validation of autophagy-related genes in keratoconus and their correlation with immune infiltration
Source: Medicine (Baltimore). 2026 May 29;105(22):e48985. doi: 10.1097/MD.0000000000048985 (PMC13225602; doi:10.1097/MD.0000000000048985)
Supplement: Supplementary file 5 [file medi-105-e48985-s006.docx]

| Description | setSize | NES | pvalue | p.adjust | rank |
| --- | --- | --- | --- | --- | --- |
| WP_GLOBO_SPHINGOLIPID_METABOLISM | 19 | 1.8931 | 0.0022 | 0.0127 | 3448 |
| MOOTHA_GLYCOGEN_METABOLISM | 17 | 1.8323 | 0.0043 | 0.0216 | 2351 |
| HOLLERN_EMT_BREAST_TUMOR_DN | 116 | 1.7700 | 0.0029 | 0.0159 | 2134 |
| KEGG_N_GLYCAN_BIOSYNTHESIS | 46 | 1.7274 | 0.0025 | 0.0144 | 3686 |
| PID_FANCONI_PATHWAY | 43 | 1.6554 | 0.0074 | 0.0327 | 4082 |
| ZHENG_IL22_SIGNALING_DN | 28 | 1.9767 | 0.0023 | 0.013 | 1967 |

Supplementary Table 5. GSEA analysis
