# Supplementary material for: Identification and validation of autophagy-related genes in keratoconus and their correlation with immune infiltration
Source: Medicine (Baltimore). 2026 May 29;105(22):e48985. doi: 10.1097/MD.0000000000048985 (PMC13225602; doi:10.1097/MD.0000000000048985)
Supplement: Supplementary file 6 [file medi-105-e48985-s007.docx]

Supplementary Table 6. GSVA analysis

| Description | logFC | AveExpr | P.Value | adj.P.Val |
| --- | --- | --- | --- | --- |
| MARIADASON_RESPONSE_TO_BUTYRATE_CURCUMIN_SULINDAC_TSA_2 | 1.0460 | -0.1240 | 0.0000 | 0.0000 |
| KUWANO_RNA_STABILIZED_BY_NO | 1.0428 | -0.0941 | 0.0000 | 0.0001 |
| GO_CYCLIC_NUCLEOTIDE_METABOLIC_PROCESS | 1.0137 | -0.1495 | 0.0000 | 0.0000 |
| GAUTSCHI_SRC_SIGNALING | 0.9742 | -0.0642 | 0.0000 | 0.0005 |
| FARDIN_HYPOXIA_9 | 0.9668 | -0.0764 | 0.0000 | 0.0002 |
| GO_REGULATION_OF_THYROID_HORMONE_MEDIATED_SIGNALING_PATHWAY | 0.9628 | -0.0493 | 0.0000 | 0.0007 |
| GO_CHONDROBLAST_DIFFERENTIATION | 0.9595 | -0.0523 | 0.0001 | 0.0030 |
| CHASSOT_SKIN_WOUND | 0.9488 | -0.0750 | 0.0000 | 0.0003 |
| GESERICK_TERT_TARGETS_DN | 0.9436 | -0.0882 | 0.0000 | 0.0001 |
| REACTOME_TRANSPORT_OF_NUCLEOTIDE_SUGARS | -0.8334 | 0.0731 | 0.0000 | 0.0002 |
| GO_CARDIOLIPIN_ACYL_CHAIN_REMODELING | -0.8353 | 0.0742 | 0.0000 | 0.0004 |
| GO_MEMBRANE_RAFT_LOCALIZATION | -0.8392 | 0.0637 | 0.0000 | 0.0007 |
| GO_MYOFIBROBLAST_DIFFERENTIATION | -0.8660 | 0.0134 | 0.0001 | 0.0023 |
| GO_REGULATION_OF_MYOFIBROBLAST_DIFFERENTIATION | -0.8660 | 0.0134 | 0.0001 | 0.0023 |
| GO_DE_NOVO_PYRIMIDINE_NUCLEOBASE_BIOSYNTHETIC_PROCESS | -0.8826 | 0.0637 | 0.0000 | 0.0003 |
| GO_AP_4_ADAPTOR_COMPLEX | -0.9145 | 0.0724 | 0.0000 | 0.0005 |
| GO_CIA_COMPLEX | -0.9158 | -0.0026 | 0.0009 | 0.0114 |
| GO_MEMBRANE_RAFT_DISTRIBUTION | -0.9416 | 0.0879 | 0.0000 | 0.0004 |
| GO_BENZODIAZEPINE_RECEPTOR_BINDING | -0.9773 | 0.0597 | 0.0000 | 0.0002 |
